# Antimicrobial Peptides: Insights into Membrane Permeabilization, Lipopolysaccharide Fragmentation and Application in Plant Disease Control

**DOI:** 10.1038/srep11951

**Published:** 2015-07-06

**Authors:** Aritreyee Datta, Anirban Ghosh, Cristina Airoldi, Paola Sperandeo, Kamal H. Mroue, Jesús Jiménez-Barbero, Pallob Kundu, Ayyalusamy Ramamoorthy, Anirban Bhunia

**Affiliations:** 1 Department of Biophysics, Bose Institute, P-1/12 CIT Scheme VII (M), Kolkata 700 054, India; 2 Department of Biotechnology and Biosciences, University of Milano-Bicocca, P.zza della Scienza 2, 2016 Milano, Italy; 3 Biophysics and Department of Chemistry, University of Michigan, 930 N. University Avenue, Ann Arbor, Michigan 48109-1055, USA; 4 Infectious Diseases Program, CIC bioGUNE, Parque Tecnologico de Bizkaia, Building 801A, 48160 Derio, Spain; 5 IKERBASQUE, Basque Foundation for Science, 48011 Bilbao, Spain; 6 Division of Plant Biology, Bose Institute, P-1/12 CIT Scheme VII (M), Kolkata 700 054, India

## Abstract

The recent increase in multidrug resistance against bacterial infections has become a major concern to human health and global food security. Synthetic antimicrobial peptides (AMPs) have recently received substantial attention as potential alternatives to conventional antibiotics because of their potent broad-spectrum antimicrobial activity. These peptides have also been implicated in plant disease control for replacing conventional treatment methods that are polluting and hazardous to the environment and to human health. Here, we report *de novo* design and antimicrobial studies of VG16, a 16-residue active fragment of Dengue virus fusion peptide. Our results reveal that VG16KRKP, a non-toxic and non-hemolytic analogue of VG16, shows significant antimicrobial activity against Gram-negative *E. coli* and plant pathogens *X. oryzae* and *X. campestris*, as well as against human fungal pathogens *C. albicans* and *C. grubii*. VG16KRKP is also capable of inhibiting bacterial disease progression in plants. The solution-NMR structure of VG16KRKP in lipopolysaccharide features a folded conformation with a centrally located turn-type structure stabilized by aromatic-aromatic packing interactions with extended N- and C-termini. The *de novo* design of VG16KRKP provides valuable insights into the development of more potent antibacterial and antiendotoxic peptides for the treatment of human and plant infections.

The remarkable increase in multi-drug resistance against conventional antibiotics observed in various pathogenic microorganisms has become one of the major concerns towards human health and global food security^1^,^2^. Several Gram-negative bacterial strains are resistant towards multiple antibiotics and pose a great threat due to the absence of active bactericidal compounds^3^,^4^. The use of antimicrobial peptides (AMPs) as novel antibiotics has been proposed and widely accepted for a long time. Due to their rapid and broad spectrum of antimicrobial properties along with their generalized mode of action, AMPs have been proposed for the treatment of microbial infections, specifically those caused by antibiotic-resistant bacteria^5^,^6^,^7^. AMPs are generally small peptides having antimicrobial activity despite a high degree of variability in their sequence, mass, charge and three-dimensional structure^8^. They constitute a vast group of molecules that are widely distributed throughout nature^9^. A variety of organisms, ranging from invertebrates to plants, animals and humans, produce AMPs to protect themselves against infection, and share common elements in their defense mechanisms against pathogens^6^. In fact, AMPs are less susceptible to fall prey to bacterial resistance than traditional antibiotics^10^. A majority of these AMPs are cationic and selectively bind to the negatively charged lipids of bacterial membrane, mainly through an electrostatic interaction, and have the ability to follow an amphipathic arrangement, with a segregation of the charged face from a hydrophobic face that permits its entry into the hydrophobic microbial membrane, leading to membrane disruption and cell death^11^,^12^,^13^. In case of Gram-negative bacteria, AMPs have to encounter lipopolysaccharide (LPS), a major component present in leaflet of the outer membrane, in order to gain access into the plasma membrane^14^,^15^,^16^. LPS acts as an efficient barrier against entry of antibiotics or antimicrobial proteins or peptides rendering them inactive; the observed resistance in Gram-negative bacteria may therefore be attributed fairly to LPS, although other modes of AMP resistance do exist^6^. A number of recent studies have demonstrated that bacterial resistance to cationic AMPs might occur through a variety of mechanisms, including chemical modification of membrane lipids, repulsion via modification of negative charges in their membrane, sequestration, proteolytic destruction, export through efflux pumps, uptake and destruction via transporters, and release of glycosaminoglycans (GAGs), polysaccharides and other polyanionic scavenging species^17^,^18^,^19^,^20^.

A major concern to global food security involves the significant worldwide loss in crops caused by plant pathogens such as bacteria, viruses, fungi and other microbial organisms; such losses account for more than 10% of the overall loss in global food production^21^. Due to their genetic variability and ability to mutate, plant pathogens continuously invade plants and compromise their tendency for growth and reproduction. Prevention and control of bacterial and fungal diseases in plants is largely based on copper compounds and other synthetic chemicals, which are considered to be environmental pollutants and may be toxic or even carcinogenic^22^. Consequently, the development of non-toxic and non-polluting treatments to control bacterial and fungal diseases in plants has been the focus of extensive research in agriculture. In this regard, non-cytotoxic membrane-associated peptides with LPS-binding affinities have attracted considerable attention as promising antibiotics for agricultural applications and plant disease control. In this study, we have investigated the antimicrobial properties of VG16, a 16 residues conserved fusion peptide chiefly responsible for host endosomal membrane fusion with viral envelope and subsequent progression of infection (Fig. 1A–C)^23^. The structural and functional characterization of the interaction of VG16 with different model membranes, such as zwitterionic dodecylphosphocholine (DPC) detergent micelles,  1-palmitoyl-2-oleoyl-sn-glycero-3-phosphocholine  (POPC)/1-palmitoyl-2-oleoyl-sn-glycero-3-phosphatidyl glycerol (POPG) lipid vesicles and anionic sodium-dodecyl-sulfate (SDS) detergent micelles, have shown that VG16 forms a loop-like structure in both neutral DPC/POPC and anionic POPG membranes^23^. A close inspection of the three-dimensional structure determined by NMR spectroscopy reveals that the structure is stabilized by a hydrophobic triad formed by Trp101, Leu107 and Phe108 of VG16 (Fig. 1B)^23^. This hydrophobic packing interaction is very crucial for membrane fusion. For instance, replacement of Trp101 with Ala eliminated the hydrophobic triad formation and completely abolished membrane fusion^23^. Since anionic membrane mimetic models, such as SDS micelles and POPG vesicles, are bacterial membrane mimetic models, the loop-like structure motivated us to utilize VG16 peptide as a building block for the *de novo* design of antimicrobial peptides against Gram-negative bacteria. In this study we show that VG16KRKP, a 16 residues analogue of VG16, exhibits a ~10-fold reduction in the MIC values against a range of Gram-negative bacteria (Fig. 1D). We also report live-cell NMR study of this peptide and attempt to provide a correlation between the three-dimensional solution structure of designed AMPs in lipopolysaccharide (LPS) (mimics the outer-membrane of Gram-negative bacteria) and its direct application to treat pathogenic bacterial infection in rice and cabbage, caused by *Xanthomonas oryzae* and *Xanthomonas campestris*, respectively. Our findings indicate that the designed peptide is capable of resisting disease progression in plants.

## Results and Discussion

### Evaluation of antimicrobial activities of rationally designed AMPs

**Figure 1:**
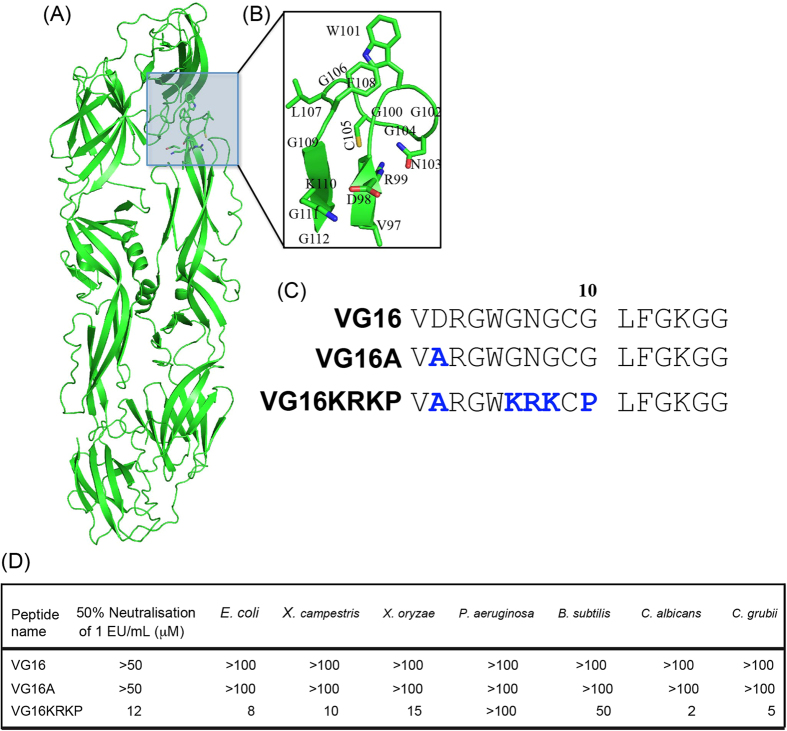
Rational design of peptides. (**A**) X-ray crystal structure of the Dengue virus envelope protein (1OAN.pdb). (**B**) Active fragment of the virus fusion peptide, VG16. (**C**) The amino acid sequences of the designed peptides VG16A and VG16KRKP. (**D**) LPS (1 EU/ml) neutralization and corresponding MIC values (in μM) against Gram-positive and Gram-negative bacteria and fungi for the peptides used in this study.

Several crystal structures of LPS-binding receptors, co-crystallized with LPS, have shown that several positively charged amino acid residues are required to stabilize the complex structure through the formation of plausible salt bridges and/or hydrogen bonds between LPS phosphate groups and protein basic residues^24^. Therefore, a high positive charge may also be vital for overcoming the anionic LPS barrier. In fact, the structured LPS-binding motif of YW12, a potent AMP designed on the basis of the structure of a β-barrel outer membrane protein of *E. coli* (FhuA) co-crystallized with LPS, comprises a centrally located stretch of four consecutive Lys and Arg residues^14^,^25^. This stretch shows multiple hydrogen bonds and salt bridge interactions with the biphosphate groups of lipid A in LPS^25^,^26^. However, inspection of the amino acid sequence of VG16 revealed a paucity of positively charged residues (Fig. 1C), responsible for electrostatic interaction between peptide and anionic LPS that enable the cell-mediated uptake of the AMPs into the hydrophobic interior. Thus, we hypothesized that inserting cationic “KRK” stretch in the VG16 peptide would improve its potency against Gram-negative bacteria (Fig. 1C). To this end, we designed VG16KRKP, where Arg and Lys residues were introduced in the extended loop region observed in the NMR structure of VG16^23^. Moreover, Pro10 was also inserted in the central region to bring hydrophobic and aromatic residues, such as Leu11 and Phe12, close to Trp5 (Fig. 1C). Interestingly, VG16KRKP is capable of neutralizing LPS by around 50% at a concentration of 12 μM (Fig. 1D). VG16 alone, without the KRKP residue, showed neither any bactericidal effect nor antifungal activity against the strains tested up to a concentration of 100 μM (Fig. 1D). Regarding the bacterial selectivity, VG16KRKP showed MIC values of 8 μM for *E. coli*, but no activity against *P. aeruginosa*, indicating the peptide is highly selective, even if both are Gram-negative bacteria. This may be attributed in part to the presence of an alginate capsule present outside the bacterial membrane in the case of *P. aeruginosa,* which is known to inhibit the entry of antimicrobial agents, rendering them inactive^27^,^28^. Nonetheless, further studies are additionally needed to investigate the presence of other potential modes of action, if any, of the designed peptide. VG16KRKP was active against plant pathogens *X. campestris* and *X. oryzae,* with comparable MIC values (Fig. 1D). It also inhibited the growth of *B. subtilis* with an MIC value of 50 μM. Moreover, VG16KRKP also showed strong antifungal activity against *Candida albicans* and *Cryptococcus grubii* with MIC values of 2 and 5 μM, respectively (Fig. 1D). In all cases, VG16 and VG16A are inactive, suggesting the importance of the presence of positive charges in the amino acid sequence. Studies on the effects of the net positive charge, hydrophobicity and amphipathicity on the activity of AMPs have shown that an increase in positively charged residues and hydrophobicity up to a certain extent while maintaining amphipathicity have led to an increase in their observed antimicrobial activity and bacterial cell selectivity^29^,^30^. In light of these results, our further studies focused exclusively on the VG16KRKP peptide.

### Live-cell NMR spectroscopy provides information on the disruption of bacterial membrane leading to cell lysis

**Figure 2:**
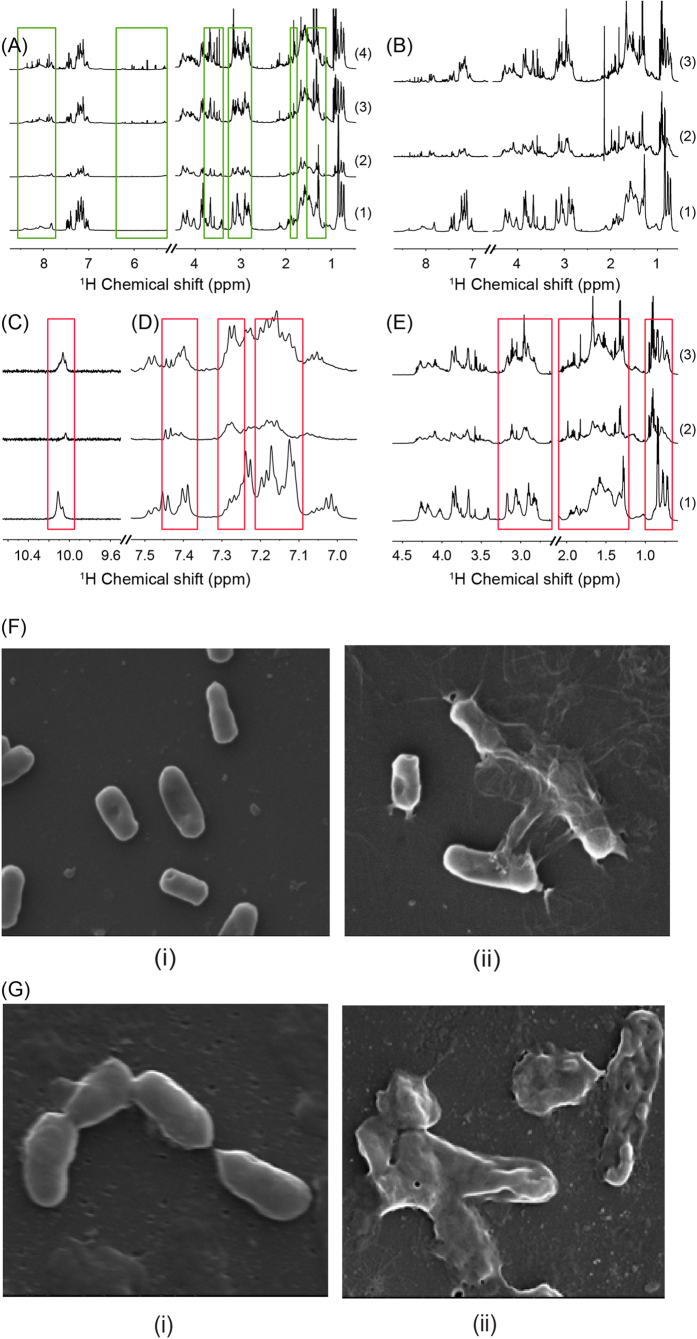
Cell lysis by VG16KRKP. (**A**) ^1^H NMR spectrum of a solution of 1.5 mM VG16KRKP, 10 mM PBS, pH 7.2 in the absence (1) or in the presence of 10^9^ cells after 30 min (2), 3 h (3) or 9 h (4) of co-incubation. Spectral regions characterized by the appearance of new signals are highlited in green. (**B**) Changes in broadening and intensity of VG16KRKP resonances after cell addition and cell dilution. (1) ^1^H NMR spectrum of a solution containing 1 mM VG16KRKP, 10 mM PBS, pH 7.2, 64 scans; (2) ^1^H NMR spectrum of a solution containing 1 mM VG16KRKP and 10^9^ cells, 10 mM PBS, pH 7.2, 64 scans (2× in intensity); (3) ^1^H NMR spectrum of a solution containing 1 mM VG16KRKP and 3.3×10^9^ cells, 10 mM PBS, pH 7.2, 64 scans (2× in intensity). The last sample was obtained by 1:3 dilution of the sample in 2 with a 1 mM VG16KRKP solution. (**C**–**E**) Chemical shift difference of VG16KRKP ^1^H resonances after cell addition and cell dilution, evidenced by expansions of spectra depicted in panel D. (**C**) NεH resonace of Trp (spectrum 1, 4× intensity; spectra 2 and 3, 8× intensity); (**D**) aromatic resonances around 7 ppm (spectrum 1, 2× intensity; spectra 2 and 3, 4× intensity); (**E**) Hα and aliphatic region (spectrum 1, 1× intensity; spectra 2 and 3, 2× intensity). All spectra were acquired on 600 MHz at 25 °C. (**F**) SEM images of *E. coli* in the (i) absence and (ii) presence of 10 μM of VG16KRKP. (**G**) SEM images of *X. oryzae* in the (i) absence and (ii) presence of 10 μM of VG16KRKP. All images were taken 45 min post incubation at 25× magnification.

Interaction of the designed VG16KRKP peptide with *E. coli* (DH5α) cell was investigated at different peptide concentrations as well as with different peptide to cell ratios using solution NMR spectroscopy. Under all employed experimental conditions, the cells started to die rather immediately after peptide addition, as evidenced from the appearance of new peaks corresponding to the metabolites released from the cells lysis (Fig. 2A). In particular, for the untreated cells, after overnight incubation, the number of vital cells was comparable with those at t_0_, while for those treated with the peptide, typically a reduction of 1 to 2 orders of magnitude in the number of colony forming units (CFU) was observed (data not shown). These data represent a further demonstration of antibacterial activity of the peptide.

One-dimensional ^1^H NMR spectra reveal dramatic broadening as well as reduction of NMR signal intensities of VG16KRKP even in the presence of different number of cells. It is worth mentioning that the concentration of the peptide was kept unchanged while the number of cells was decreased by a factor of 2, 3 or 4, depending on the dilution factor (Fig. 2B). After several hours of co-incubation, the peptide resonance intensities considerably increased, while the line shape returned to a stage comparable to those of the peptide alone, as a consequence of significant cell death and subsequent peptide dissociation (Fig. 2A). The interaction could also be deduced from the dramatic changes in the indole (NεH) ring protons of Trp5 (resonating at ~10 ppm) (Fig. 2C), aromatic resonances (Fig. 2D), along with methyl and other aliphatic protons (Fig. 2E) of VG16KRKP.

Furthermore, scanning electron microscopy (SEM) was performed to determine the rate of killing of the bacteria by VG16KRKP. Bacterial suspension of the two Gram-negative bacteria E*. coli* and *X. oryzae,* containing 10^6^ cells, were incubated with VG16KRKP for different time intervals and analyzed by SEM in order to understand the nature and extent of cell lysis (Supplementary Fig. S1). The concentration of VG16KRKP used was close to MIC against both the Gram-negative bacteria (Fig. 1D). Interestingly, shrinkage in the bacterial wall and cell lysis, leading to leakage of intracellular material, was evident from SEM images as early as 5 min post cell incubation with the peptide (Supplementary Fig. S1). After 45 min of incubation, no clear shape for cells was observed (Fig. 2F,G), indicating that the peptide is very active and efficient against both the Gram-negative bacteria used here.

### VG16KRKP binds LPS, which in turn mediates its disaggregation

**Figure 3:**
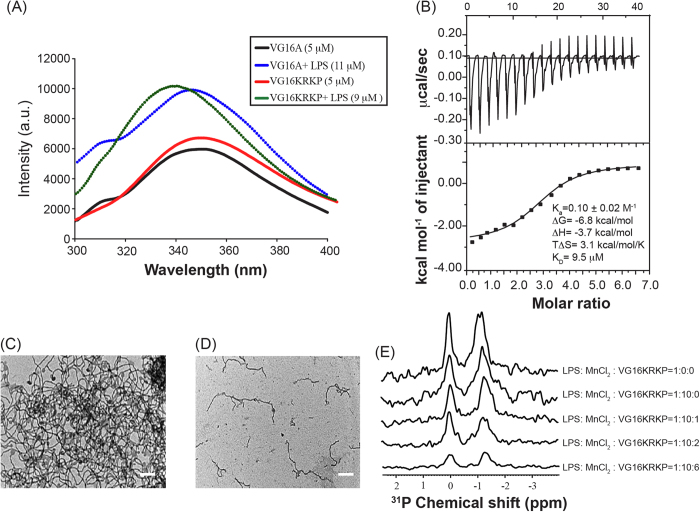
Binding studies of VG16KRKP with LPS. (**A**) Intrinsic tryptophan fluorescence emission spectra of VG16A or VG16KRKP in the absence and presence of LPS micelle. (**B**) Upper panel showing endothermic heat of reaction vs. time (minute) upon interaction of VG16KRKP with LPS micelle. The lower panel shows enthalpy change per mole of peptide injection vs. molar ratio (peptide:LPS) for VG16KRKP. In this experiment, 50 μM of LPS micelle was titrated against 200 μM of peptide, VG16KRKP. TEM images of LPS micelle (**C**) alone and (**D**) in the presence of VG16KRKP. Scale bar = 1 μm. (**E**) ^31^P NMR spectra of the phosphates of LPS micelle in presence of MnCl_2_ in free form and upon addition of increasing concentrations of VG16KRKP signifying the fragmentation of LPS micelles, as evident from the reduction in intensity.

As mentioned earlier, AMPs should first interact with LPS before gaining access into the cell for its lysis. The intrinsic fluorescence of the Trp residue present in the peptides was used to determine the binding parameters. Addition of small aliquots of LPS into the sample containing VG16/VG16A did not show significant blue shift (~3 nm) of Trp fluorescence (Fig. 3A). In contrast, ~11 nm of blue shift was observed in the emission maxima of VG16KRKP upon successive addition of LPS (Fig. 3A). The noticeable blue shift of the emission wavelength is a strong evidence of the insertion of the Trp residue of VG16KRKP into the LPS hydrophobic environment. Additionally, downward trends of the ITC profiles were observed for the binding interaction of either VG16A or VG16KRKP with LPS, suggesting an exothermic or enthalpy-driven process where electrostatic/ionic interaction plays a vital role. Figure 3B and Supplementary Fig. S2 summarize the thermodynamic parameters of peptide binding to LPS. The interaction of VG16KRKP with LPS has been estimated to have dissociation constant (K_D_) of 9.5 μM, one order of magnitude lower than that for VG16A (Supplementary Fig. S2). Taken together, these results suggest that the lack of positive charges in VG16/VG16A impedes their efficient binding to the LPS micelle.

To further explore the bacterial entry process through LPS layer, a combination of spectroscopic and microscopic methods was utilized. Transmission electron microscopy (TEM) images of LPS obtained in the absence and in the presence of VG16KRKP are shown in Fig. 3C,D, respectively. LPS in aqueous solution shows a ribbon-like assembly with variable width, thickness and few hundred μm length (Fig. 3C). This result indicates the formation of large inhomogeneous aggregation of LPS. A similar observation has been reported earlier in two independent studies^31^,^32^. In contrast, TEM images confirmed the disaggregation of ribbon-like assembly of LPS to small thread-like structures with filamentous forms in the context of VG16KRKP treatment for 3 hours (Fig. 3D). In addition, small dense spherical particles of LPS molecules in the presence of VG16KRKP were also observed from the TEM image (Fig. 3D). Similar morphological changes of LPS in the presence of the KYE28 peptide (derived from human heparin cofactor II) have been recently observed^33^. Shai and co-workers have also reported EM images of LPS upon treatment with a series of 12 amino-acid peptides and their fatty acid conjugated analogues to study disaggregation^29^. Similar conclusions can also be drawn from dynamic light scattering (DLS) experiments. The hydrodynamic diameter (~1000 nm) and high polydispersity of LPS in aqueous solution show two- and seven- fold decrease upon incubation with VG16 and VG16KRKP, respectively (Supplementary Fig. S3). This result also supports that VG16KRKP has a stronger effect on disaggregation of LPS micelle. Studies of LPS disaggregation using light scattering studies demonstrating a reduction in polydispersity and diameter of LPS micelles upon treatment with AMP have been previously reported^34^.

In order to gain more insights into the mechanism of disruption of LPS aggregation at atomic-resolution, ^31^P NMR experiments of LPS alone as well as in the presence of different concentrations of VG16KRKP were carried out using MnCl_2_, a paramagnetic quencher, as a dopant. The paramagnetic ion Mn^2+^ quenches ^31^P NMR peaks of LPS phosphate head groups in its vicinity. In the absence of VG16KRKP, a negligible quenching of the phosphate head group signal was observed for the sample containing 10 mM MnCl_2_ and 0.5 mM LPS (Fig. 3E). The heterogeneous aggregation of LPS makes Mn^2+^ ions inaccessible to the phosphate groups of LPS. Addition of VG16KRKP to the sample containing LPS at a molar ratio of 1:1 showed a negligible effect on ^31^P peaks of LPS phosphate groups, confirming that LPS remains intact as a heterogeneous aggregate (Fig. 3E). However, upon subsequent addition of up to 3 mM VG16KRKP (LPS:VG16KRKP = 1:6), a drastic quenching of the intensity of ^31^P peaks of LPS phosphate head groups was detected. This result points towards the fragmentation or disruption of LPS aggregation by formation of a small lipid vesicle, which tumbles sufficiently fast on the NMR time scale (Fig. 3E), suggesting that the peptide follows detergent-like mechanism to fragment LPS aggregates. Collectively, the results from ^31^P NMR experiments on LPS in the presence of the MnCl_2_ quencher support the hypothesis of a two-step mechanism of membrane fragmentation demonstrated for AMP or amyloid beta peptide^35^,^36^.

### Structural insights in the absence and presence of LPS by NMR spectroscopy

**Figure 4:**
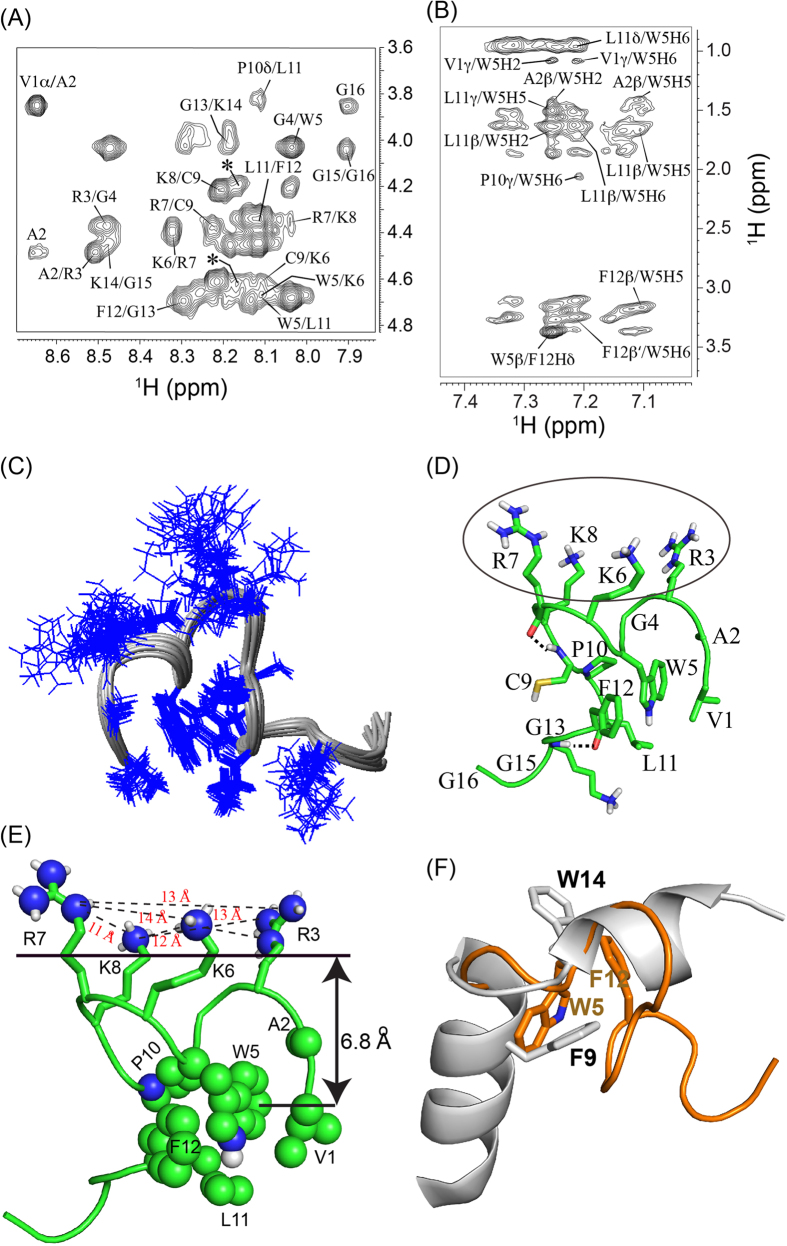
Structure of VG16KRKP in LPS. Selected regions of two-dimensional ^1^H-^1^H trNOESY spectra of VG16KRKP in LPS showing (**A**) fingerprint region of C^α^H-NH resonances, and (**B**) long-range NOEs between aromatic ring protons and aliphatic side chain residues. Peaks, which are marked by the symbol * are unassigned due to the *cis-*trans configuration at Cys9-Pro10 bond. All experiments were performed on a Bruker Avance 500 MHz at 25 °C. (**C**) Side chain representation of twenty-ensemble VG16KRKP structures in LPS. (**D**) Cartoon representation of average structure of VG16KRKP conformations bound to LPS. The hydrogen bonds, which help in the stabilization of structures, are shown as black dotted lines. All the positive charges are facing one side, marked by circle. (**E**) Hydrophobic packing constituted by the residues Val1, Ala2, Trp5, Pro10, Leu11 and Phe12 are shown by space filling. Interestingly, the positive charge residues maintain a distance similar to the distance between two phosphate head groups of LPS (~12 Å). Depth of insertion study using fluorescence quencing experiments show that the position of Trp5 residue is ~6.8 Å from the center of the LPS bilayer, suggesting the Trp and othe residues of VG16KRKP, associated with Trp have strong van-der-Waals interaction with the acyl chain of LPS. (**F**) Superposition of DPC bound fusion domain of hemagglutinin (1IBN.pdb) (structure is stabilized by i, i + 5 residues) and LPS bound VG16KRKP (2 MWL.pdb) (structure is stabilized by i, i + 7 residues).

One-dimensional ^1^H NMR spectrum was monitored to understand the binding of peptides to LPS. Addition of small but increasing concentrations of LPS caused visible concentration-dependent broadening (without inducing any chemical shift change) for most of the proton resonances of VG16A as well as those of VG16KRKP (Supplementary Fig. S4), implying a fast chemical exchange between free and bound forms of the peptide in the NMR time scale, which is an ideal situation to determine the bound conformation of the peptide in the presence of LPS by transferred NOESY (trNOESY)^37^,^38^. It is worth mentioning that LPS aggregates into a large molecular weight micelle/bilayer at 14 μg/mL concentration^39^. The trNOESY spectra of VG16 (Supplementary Fig. S5A, left panel) and VG16A (Supplementary Fig. S5A, right panel) showed very few cross peaks characterized by intra-residual as well as sequential NOE contacts between the backbone and side-chain proton resonances (Supplementary Fig. S5). It is interesting to note that 43.8% of the residues of VG16/VG16A are Gly and hence, due to its flexibility, VG16/VG16A are highly dynamic in aqueous solution as well as in LPS (Supplementary Fig. S5D). On the other hand, the trNOESY spectra of VG16KRKP at a LPS:peptide molar ratio of 1:50 yielded a large number of NOE cross peaks, thus signifying the development of a well-folded conformation (Fig. 4A,B). Analysis of the spectra revealed the presence of strong sequential αN (i, i + 1) and HN/HN NOEs for most of the residues along with few long range (i to ≥ i + 5) NOEs. A closer look at the NOE distribution showed that residues Val1, Ala2, Trp5, Cys9, Pro10, Leu11 and Phe12 were characterized by a higher number of NOE contacts in the presence of LPS (Fig. 4B and Supplementary Fig. S6). All long-range NOE contacts are summarized in Table S1. The most important long-range NOE contacts were observed between the ring protons of Trp5 and the aliphatic side-chain (β, γ and δ) protons of Leu11. NOE contacts were additionally observed between the residues Trp5 and Phe12 (Fig. 4B). Surprisingly, the indole (NεH) ring protons of Trp5 did not show any NOE contact with other peptide residues. The Cys9-Pro10 bond of VG16KRKP in LPS adopts *trans* conformation due to the presence of Cys9CαH/Pro10CδHs NOEs. Additionally, several long range NOEs such as Phe11CδHs/Trp5CβHs, Trp5H6/Leu11CαH, Cys9CαH/Lys6, Lys14CαH/Leu11 and Pro10CγHs/Trp5H6 are also observed (Fig. 4B and Table S1). Notably, the αN (i, i + 1) NOEs such as Trp5/Lys6 and Arg7/Lys8 are broad in nature (Fig. 4A), indicating the dynamic properties of “KRK” segment of VG16KRKP. Strikingly, the C-terminal residues Gly13-Lys14-Gly15-Gly16 of VG16KRKP did not show any NOEs in the context of LPS, indicating that this region still remains highly flexible.

### Three-dimensional structure of VG16KRKP in LPS

Twenty ensemble structures of VG16KRKP associated to LPS was determined using NOE based distance constraints (Fig. 4C,D and Table 1) and verified using PROCHECK NMR^40^. The LPS-bound backbone ensemble structure of VG16KRKP was rigid whereas the side chains of the positively charged residues remain highly dynamic. The positively charged ammonium (H_3_N^+^-) group of Lys residues and guanidinium groups of Arg residues of VG16KRKP maintain a distance of ~11–14 Å (Fig. 4E), comparable to that obtained between the two phosphate head groups of the lipid A moiety of LPS^41^. The structure of LPS-bound VG16KRKP is amphipathic, with the positively charged residues (Arg3, Lys6, Arg7 and Lys8) oriented in one specific direction, thus forming a charged surface region (Fig. 4D,E). Conversely, the hydrophobic residues Trp5, Leu11 and Phe12 from the central region of the peptide sequence pack together forming a hydrophobic triad, and stabilize a loop-type structure (Fig. 4D,E). This hydrophobic cluster is further intensified by the presence of Val1 and Ala2, which are packed towards Trp5, and by Pro10 (Fig. 4E). Due to the lack of NOEs, the C-terminus, Gly13-Lys14-Gly15-Gly16, is extended (Fig. 4E). Interestingly, this structure bears a close resemblance to the LPS-bound structure of the synthetic peptide YI12, a modified and more potent form of YW12. This peptide and the fusion domain of the influenza virus haemagglutinin protein in DPC micelles show i to i + 5 aromatic packing interactions between Phe and Trp residues (Fig. 4F)^42^; they resemble the i to i + 7 aromatic packing interaction between Trp5 and Phe12 observed herein. The position of Trp residue of VG16KRKP in the hydrophobic core of LPS bilayer was measured using fluorescence quenching experiments in the presence of two spin-labeled fatty acids, 5-DSA (5-doxyl stearic acid) and 16-DSA (16-doxyl stearic acid). It was found that the Trp residue of VG16KRKP was around 6.8 Å from the center of the LPS bilayer (Fig. 4E), suggesting that the Trp residue as well as the associated hydrophobic hub are inserted into the hydrophobic core of LPS bilayer, most likely interacting with the acyl chains of LPS.

### VG16KRKP is non-toxic and non-hemolytic

**Figure 5:**
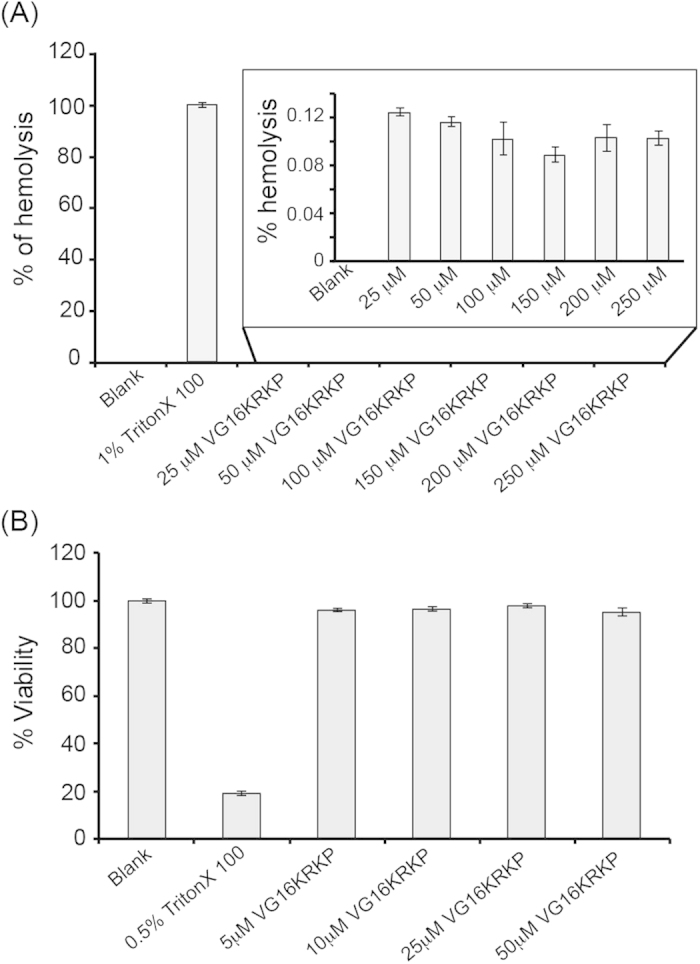
Hemolytic and cytotoxic effects of VG16KRKP. (**A**) Bar plot showing percentage of hemolysis of human RBC upon addition of increasing concentrations of VG16KRKP. (**B**) Bar plot showing percentage viability of human fibrosarcoma cell line upon treatment with increasing concentrations of VG16KRKP.

To evaluate VG16KRKP as a therapeutic agent, we performed hemolytic assay on human blood samples and cytotoxicity assay on HT1080 cell line. The *in vitro* hemolytic assay on human blood measures the hemoglobin release in the plasma as a consequence of RBC lysis mediated by the agent being tested. Interestingly, VG16KRKP showed almost no hemolysis of RBC up to a concentration of 250 μM, ~30 times higher than its MIC value (Fig. 5A), whereas 2% Triton X, used as a control, did 100% of hemolysis. Furthermore, VG16KRKP did not show any significant (less than 5%) toxicity on HT1080 cell line up to a final concentration of 50 μM VG16KRKP, i.e., ~6 times higher than the MIC value (Fig. 5B). The 0.5% Triton X 100 was used as a control for the toxicity assay and it showed only 20% cell viability after treatment with Triton X 100. These results collectively indicate that VG16KRKP is a non-hemolytic and non-toxic peptide.

### VG16KRKP-treated *Xanthomonas* shows impaired infectivity to plant

**Figure 6:**
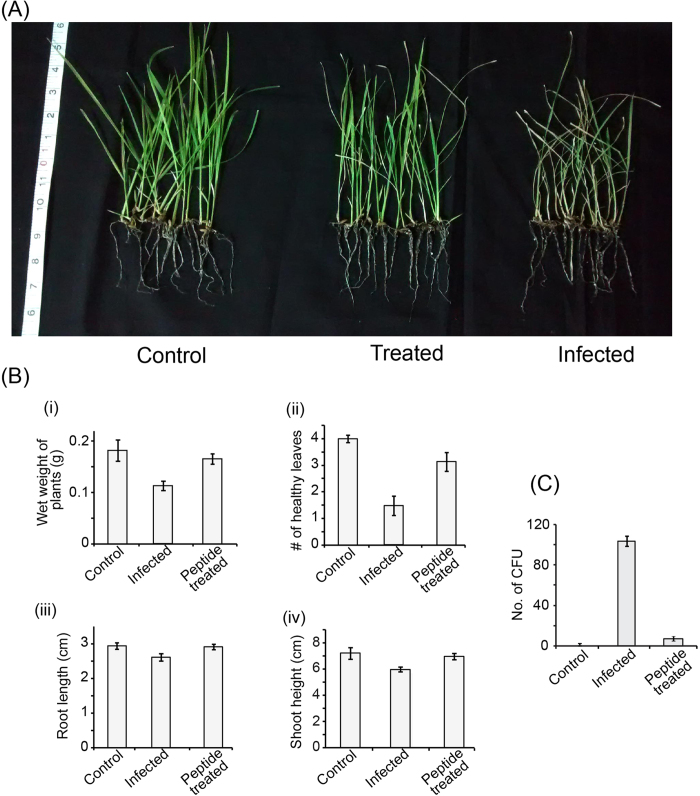
Application of VG16KRKP in treating *X. oryzae* infection in rice plants. (**A**) Images of 67 days old uprooted control, infected, and peptide-treated rice plants showing inhibition of disease upon treatment. (**B**) Bar plots showing variation in wet weight of plants (i), healthy leaves (ii), root length (iii), and shoot height (iv) between control, infected and peptide-treated rice plants. A student’s t-test was performed in each case. (**C**) Bar plot of number of colony forming units (CFU) of *X. oryzae* obtained from equal amounts of crushed leaf tissue of control, infected, and peptide-treated plants which show a 10-fold reduction in the number of colonies in treated plants, confirming that VG16KRKP is capable of inhibiting disease.

Our data showed significant antimicrobial activity against two devastating plant pathogens, namely *Xanthomonas oryzae* and Xanthomonas *campestris* (Fig. 1D), isolated from the fields of Kalyani, West Bengal, India. To depict the efficiency of the peptide in inhibiting leaf blight disease development *in vivo*, the *in vitro* mixtures used for the antimicrobial assays were also used to inoculate rice plants. *X. oryzae* alone and the bacteria pretreated with 500 μM VG16KRKP were used for inoculation. Leaf curling was observed in 86% of infected plants, 5 days post infection, and also to a greater extent when compared to that observed in only peptide treated plants (28% had any disease-like symptom) (Fig. 6). At 10 to 12 days post infection, lesion formation was also more pronounced in infected plants compared to peptide treated plants. In control plants, no leaf curling or lesion formation was observed (Fig. 6A). Upon observation of uprooted plants, the wet weight of infected plants (n = 14 in each set) was found to be 38% lesser compared to control plants (Fig. 6B,i). The wet weight of treated plants was however only 9% lesser than the control plants (Fig. 6A,B). The number of healthy leaves was 63% and 22% lower for bacteria-infected plants and peptide-treated plants, respectively, in comparison to control plants (Fig. 6B,ii). Bacteria-infected plants root length and shoot height were slightly affected, showing a reduction of 11% and 17%, respectively. In contrast, peptide-treated plants show negligible effect with a reduction of 1% and 4%, respectively (Fig. 6A,B,iii,iv). These results indicate that peptides are capable of weakening the pathogen, thus an inhibition of disease progression has occurred.

To further quantify the pathogen in rice plants upon treatment, equal amounts of surface sterilized leaf tissues from mock infected, *Xanthomonas*-infected and peptide-treated *Xanthomonas* infected plants were crushed and plated on suitable media and *X. oryzae* growth was compared. No colonies were observed in control sets even when clarified tissue extract was spread. Samples from plants infected with peptide-treated bacteria had a ~10 fold reduction in the number of colony forming units (CFU) compared to infected samples. Similar data was also obtained when 10-fold diluted samples were used (Fig. 6C). These data indicated that VG16KRKP-treated bacteria were unable to sustain their growth in planta, thus the peptide could effectively prevent bacterial disease development in a crop plant. We also extended our study with same peptide on *X. campestris*, a causative agent of black rot infection in cabbage (*Brassica oleracea*) and observed that VG16KRKP-treated plants show almost similar symptoms as control plants (see Supplementary Fig. S7). Taken together, VG16KRKP is able to control the bacteria infected plant disease for two different plant systems. Meanwhile, it is important to mention that degradation of the peptide on the plant surface, caused by proteases and phenolic compounds, cannot be completely ruled out^43^. Nevertheless, growth promoting bacteria are generally found to be associated with the rhizosphere and therefore may be protected from exposure to peptide^44^,^45^.

Recently, the three-dimensional structure of a plant defensin antimicrobial peptide has been determined^46^. Most of the plant defensins are cysteine-rich peptides and are responsible for innate immunity and metal tolerance, such as zinc, in plants^47^,^48^. Unlike AMPs, the defensins are larger in size, and their positive charge along with their hydrophobic residues are scattered at the surface of the molecule, hence they do not adopt amphipathic structure^49^. In most of the plant defensins, Pro residue plays an important role as well, where the prolines mostly prefer *trans* conformation than *cis* conformation^46^. In general, proline rich AMPs, isolated from insects, have also been investigated extensively due to their variety of modes of action to destabilize the membrane^50^,^51^. Additionally, Pro-rich AMPs cross the blood brain barrier (BBB) easily, and hence can be used as a potential novel carrier. It is to be noted that the VG16KRKP peptide contains Cys as well as Pro residues, which means it satisfies the plant defensin activity. Moreover, the Pro residue of VG16KRKP prefers *trans* conformation in LPS. We, therefore, believe that our small designed AMP may be an alternative solution to plant defensins for killing bacteria.

## Conclusion

Unlike current methods for agricultural pathogen management that include applications of hazardous chemicals, AMPs are of paramount interest for application in agriculture. They are biodegradable and generally do not induce bacterial resistance^52^,^53^. In fact, small peptides that form a major part of defense mechanisms of a variety of organisms have been widely used for the development of genetically engineered disease-resistant plants^54^,^55^. Therefore, application of AMPs towards the protection of crops can help in controlling plant pathogens and in turn improve agriculture by reducing environmental hazards. We have provided a comprehensive study on a *de novo* designed peptide, both from structural and functional aspects, including its application for treating plant disease, thus enabling a correlation between the two aspects. Observing the potency of this peptide against Gram-negative plant pathogens such as *X. campestris* and *X. oryzae*, we have studied disease progression in rice and cabbage through external application of peptide-treated pathogens. Peptide treatment to the bacteria was effective in inhibiting diseases to a significant extent. This observation is of considerable importance, as it demonstrates the potential use of the peptide in controlling agriculturally important pathogens. Further studies will be useful to modify the peptide for increasing its potency against pathogens, and to study its stability and economical feasibility in usage as a foliar spray for inhibiting plant diseases. Our present results also encourage the development of disease resistant transgenic plants. In this context, studies involving the preparation of nanoparticles attached to AMPs for external applications to the plant and the development of transgenic plants including the overexpressed designed peptide are in progress.

## Methods

### Plant Materials and Culture

Grains of rice (*Oryza sativa*) variety IR64 were surface sterilized using 5% HgCl_2_, washed thoroughly with distilled water to remove all traces of HgCl_2,_ and planted in soil rite. The plants were grown in normal light at 30 °C. Cabbage (*Brassica oleracea*) seeds variety VC612 and Golden Acre obtained from Sutton Seeds, India, were soaked overnight in water, planted in Soil rite (Keltech, India) and grown in a growth chamber at 10000 lux, 25 °C, 85% relative humidity and a photoperiod of 16 hours.

### Antimicrobial Activity

The detailed antimicrobial activity of the peptides VG16, VG16A and VG16KRKP against *E. coli* DH5α, *X. campestris* pv. *Campestris*, *X. oryzae*, *B. subtilis*, *P. aeruginosa*, *C. albicans* and *C. grubii* is described in the Supplemental Information.

### Scanning Electron Microscopy (SEM)

*E. coli* and *X. oryzae* were cultured in luria broth (LB) and PS broth (Peptone 1%, sucrose 1%), respectively to mid-log phase and harvested by centrifugation at 8000 rpm for 10 min. Cell pellets were washed twice with 10 mM PBS and resuspended to an OD_600_ of 0.01. The cell suspensions were incubated with peptide at a concentration corresponding to its MIC for different time periods starting from 5 min to 1 hour at 37 °C. After incubation, 10 μL of bacterial suspension was spotted on a slide and fixed with 10 μL of 4% (v/v) glutaraldehyde in PBS at 4 °C overnight. Thereafter, the slides were washed twice with PBS and dehydrated by treatment with a graded ethanol series (30%, 50%, 70%, 90%, and 100%), for 15 min each. The samples were air dried, followed by gold coating and observed under a SEM.

### Transmission Electron Microscopy (TEM)

A suspension of *E. coli* 0111:B4 LPS (0.1 mg/mL) in 10 mM phosphate buffer, pH 7.4 was incubated with VG16KRKP for 3 hours in a molar ratio of 1:1. The 1 μL aliquot was placed on a formvar coated copper grid, stained with 1% uranyl acetate, dried and imaged in a JEOL 110 Operating at 80 KV voltage. An LPS sample without peptide served as a control.

### Fluorescence Spectroscopy

The intrinsic Trp fluorescence emission spectra of 5 μM peptide upon titration with increasing concentrations of LPS (ranging from 0 to 10 μM) was measured at an emission range of 300–400 nm using an excitation wave length of 280 nm, and excitation and emission slit of 5 nm. All the fluorescence experiments were performed at 25 °C in a quartz cuvette of path length 0.1 cm using a Hitachi F-7000 FL spectrometer. Peptide and LPS stocks were prepared in 10 mM Phosphate buffer of pH 6.0. The detailed method for the calculation of depth of insertion has been discussed in supplemental information.

### Isothermal Titration Calorimetry (ITC)

The binding interaction of VG16 or VG16KRKP with LPS was assessed using iTC200 micro-calorimeter (MicroCal Inc., Northampton, MA). Peptide and LPS stocks in 10 mM phosphate buffer, pH 7.4 were degassed. A 300 μL sample cell containing 50 μM LPS was titrated with the peptide from a stock solution of 1.5 mM at 298 K and a stirring speed of 300 rpm. A total of 20 injections, at an interval of 120 sec with 2 μL of peptide aliquots per injection were performed. Micro Cal Origin 5.0 software was used to plot the raw data and the association constant (K_a_), change in heat of enthalpy of (ΔH), free energy of binding (ΔG) and entropy (ΔS) were analyzed using a single site binding model using the equations ΔG = −RT ln K_a_ and ΔG = ΔH−TΔS, respectively.

### NMR Spectroscopy

All NMR experiments were performed at 25 °C on a Bruker Avance III 500 MHz NMR spectrometer, equipped with a 5 mm SMART probe. Two-dimensional (2D) homonuclear TOCSY (80 ms mixing time) and NOESY (200 ms mixing time) were acquired for 1 mM of peptide in aqueous solution with 10% D_2_O and a pH of 4.5. The spectral width was 11 ppm in both dimensions. The interaction of the peptide with LPS was monitored by 1D proton NMR spectra with a line broadening effect of the peptide (1 mM) in the presence of various concentrations of LPS. 2D NOESY experiments for each peptide (VG16/VG16A/VG16KRKP) in LPS were performed with three different mixing times (100, 150 and 200 ms). 456 t1 increments (with 112 scans and 16 dummy scans per increment) and 2 K t2 data points were recorded in each experiment with a 1s recycle delay. TOCSY and NOESY experiments were performed with States TPPI^56^ and quadrature detection in t1 dimension; WATERGATE was used for water suppression^57^. All 2D spectra were processed with a squared sine bell apodization and zero filling to 4 K (t2) × 1 K (t1) data matrices. All experiments were recorded using 4, 4-dimethyl-4-silapentane 5-sulfonate sodium salt (DSS) as an internal standard (0.0 ppm). A series of 1D proton-decoupled ^31^P NMR spectra of 0.5 mM LPS alone and upon titration with increasing concentrations of peptides, with and without 5 mM MnCl_2_ as a paramagnetic quencher, were recorded at 298 K with 3,072 scans (measurement time ~90 minutes/experiment). Both LPS and peptides were dissolved in water supplemented with 10% D_2_O at pH 4.5.

### Live-cell NMR Experiments

Live-cell NMR experiments were performed on a Bruker Avance III 600 MHz NMR spectrometer, equipped with a 5 mm QCI cryoprobe. Each sample (total volume 550 μL) was transferred into a 5 mm NMR tube and maintained at a temperature of 310 K during experiments. Final peptide concentration was in the range of 0.5–1.5 mM, with a total cell number varying from 3 × 10^8^ to 2 × 10^9^. Sample preparation and other NMR experimental parameters are given in detail in the Supplementary Information.

### Calculation of NMR Derived Structures

For the NMR-derived peptide structure calculations, the volume integrals of NOE cross-peaks were qualitatively differentiated into strong, medium and weak, depending on their intensities in the NOESY spectra in presence of either LPS. This information was further converted to inter-proton upper bound distances of 3.0, 4.0 and 5.0 Å for strong, medium and weak, respectively, while the lower bound distance was fixed to 2.0 Å. The backbone dihedral angle (phi) of the peptide kept flexible (−30° to −120°) for all non-glycine residues to limit the conformational space. The CYANA program *v2.1* was used for all structure calculations^58^ with iterative refinement of the structure based on distance violation (hydrogen bonding as constraint was excluded for structure calculation). The stereochemistry of NMR-derived ensemble structures were checked using Procheck^40^. The calculated structures have been deposited to protein data bank (PDB) with accession codes of 2 MWL.

### Antibacterial Assay on Rice and Cabbage Plants

40 days old cabbage and 55 days old rice plants were used for the assay. Three experimental sets of plants were maintained. The rice plants were inoculated by clip inoculation method^59^. Briefly, log phase cultures of the infecting bacteria were washed three times and re-suspended in 10 mM phosphate buffer, pH 7.4 to an OD of 1, roughly corresponding to 10^8^ cells/mL and were used for infection. Sterile scissors were dipped into cell suspension and leaf tips were excised. For infecting cabbage plants, an insulin needle (BD Sterile needle, 32 G × 5/32¨) was used to prick the veins of the leaves and cell suspension was applied using a cotton swab. 1 O.D. cell suspensions of *X. oryzae* and *X. campestris pv campestris* alone were used to inoculate the infected set of rice and cabbage plants respectively. Control sets of plants were inoculated with phosphate buffer (pH 7.4) alone. 1 O.D. cell suspension of *X. oryzae or X. campestris pv. campestris* pre-treated for 5 hours with 500 μM VG16KRKP were used to inoculate treated set of rice and cabbage plants respectively. The rice and cabbage plants were observed for development of symptoms up to 12 and 30 days post infection (dpi) respectively, uprooted and compared to observe morphological changes, if any.

### Determination of Pathogen Density in Plant Tissue

Leaf tissue (20 mg) was cut with individual sterile scissors from control, infected and treated sets of rice plants, surface sterilized by dipping in 5% HgCl_2_ for 5 to 7 minutes and washed well in autoclaved double distilled water to remove all traces of HgCl_2_. Sterile condition was maintained during rest of the procedure. Tissues were crushed in 1 mL 10 mM phosphate buffer, pH 7.4 using separate mortar and pestle maintained for each set. An aliquot of the suspension was diluted ten folds (up to 10^−2^ dilution). Undiluted (50 μL) and diluted tissue suspensions were spread on LB agar plates supplemented with 50 μg/mL Rifampicin in triplicates and incubated at 28 °C for 2-3 days till bacterial growth appeared. The CFU were counted in each case and compared. The experiment was repeated thrice.

### Statistics

All biological experiments were repeated at least three times and three biological replicates were used wherever applicable. Results are expressed as mean ± standard error. Student’s t-test was also performed to determine the statistical significance of the difference between two populations of plants and p value ≤ 0.05 was considered significant.

## Additional Information

**Accession codes**: The structure of VG16KRKP bound to LPS has been deposited to protein data bank (PDB) with accession number 2 MWL.

**How to cite this article**: Datta, A. *et al.* Antimicrobial Peptides: Insights into Membrane Permeabilization, Lipopolysaccharide Fragmentation and Application in Plant Disease Control. *Sci. Rep.* **5**, 11951; doi: 10.1038/srep11951 (2015).

## Supplementary Material

Supplementary Information

## Figures and Tables

**Table 1:** Summary of structural statistics for the 20 lowest energy ensemble structures of VG16KRKP in LPS.

Distance restraints	
Intra-residue (i−j = 0)	29
Sequential (|i−j| = 1)	55
Medium-range (2≤|i−j|≤4)	10
Long-range (|i−j|≥5)	10
Total	104
Angular restraints	
Φ	13
Ψ	13
Distance restraints from violations (≥0.4 Å)	0
Deviation from mean structure (Å)	
Average backbone to mean structure	0.32 ±0.09
Average heavy atom to mean structure	1.00 ±0.16
Ramachandran plot^a^	
% Residues in the most favourable and additionally allowed regions	90.5
% Residues in the generously allowed Region	9.5
% Residues in the disallowed region	0.0

^a^Procheck NMR based analysis.

## References

[b1] Levy S. B. & Marshall B. Antibacterial resistance worldwide: causes, challenges and responses. Nat Med 10, S122–9 (2004). 15577930 10.1038/nm1145

[b2] Zasloff M. Antimicrobial peptides of multicellular organisms. Nature 415, 389–95 (2002). 11807545 10.1038/415389a

[b3] Walsh F. M. & Amyes S. G. B. Microbiology and drug resistance mechanisms of fully resistant pathogens. Curr Opin Microbiol 7, 439–444 (2004). 15451497 10.1016/j.mib.2004.08.007

[b4] Taubes G. The Bacteria Fight Back. Science 321, 356–361 (2008). 18635788 10.1126/science.321.5887.356

[b5] Hancock R. E. Peptide antibiotics. Lancet 349, 418–22 (1997). 9033483 10.1016/S0140-6736(97)80051-7

[b6] Brogden K. A. Antimicrobial peptides: pore formers or metabolic inhibitors in bacteria? Nat Rev Microbiol 3, 238–50 (2005). 15703760 10.1038/nrmicro1098

[b7] Sahl H. G. Optimizing antimicrobial host defense peptides. Chem Biol 13, 1015–7 (2006). 17052605 10.1016/j.chembiol.2006.10.001

[b8] Haney E. F. & Vogel H. J. NMR of Antimicrobial Peptides. Annu Rep NMR Spectrosc 65, 1–51 (2009).

[b9] Maróti G., Kereszt A., Kondorosi E. & Mergaert P. Natural roles of antimicrobial peptides in microbes, plants and animals. Res Microbiol 162, 363–74 (2011). 21320593 10.1016/j.resmic.2011.02.005

[b10] Hancock R. E. & Patrzykat A. Clinical development of cationic antimicrobial peptides: from natural to novel antibiotics. Curr Drug Targets Infect Disord 2, 79–83 (2002). 12462155 10.2174/1568005024605855

[b11] Epand R. M. & Vogel H. J. Diversity of antimicrobial peptides and their mechanisms of action. Biochim Biophys Acta 1462, 11–28 (1999). 10590300 10.1016/s0005-2736(99)00198-4

[b12] Matsuzaki K. Magainins as paradigm for the mode of action of pore forming polypeptides. Biochim Biophys Acta 1376, 391–400 (1998). 9804997 10.1016/s0304-4157(98)00014-8

[b13] Ramamoorthy A. Beyond NMR spectra of antimicrobial peptides: Dynamical images at atomic resolution and functional insights. Solid State Nucl Magn Reson 35, 201–207 (2009). 19386477 10.1016/j.ssnmr.2009.03.003 PMC2694728

[b14] Bhunia A., Mohanram H., Domadia P. N., Torres J. & Bhattacharjya S. Designed beta-boomerang antiendotoxic and antimicrobial peptides: structures and activities in lipopolysaccharide. J Biol Chem 284, 21991–2004 (2009). 19520860 10.1074/jbc.M109.013573 PMC2755923

[b15] Snyder D. S. & McIntosh T. J. The lipopolysaccharide barrier: correlation of antibiotic susceptibility with antibiotic permeability and fluorescent probe binding kinetics. Biochemistry 39, 11777–87 (2000). 10995246 10.1021/bi000810n

[b16] Rosenfeld Y., Barra D., Simmaco M., Shai Y. & Mangoni M. L. A synergism between temporins toward gram-negative bacteria overcomes resistance imposed by the lipopolysaccharide protective layer. J Biol Chem 281, 28565–28574 (2006). 16867990 10.1074/jbc.M606031200

[b17] LaRock C. N. & Nizet V. Cationic antimicrobial peptide resistance mechanisms of streptococcal pathogens. Biochim Biophys Acta 10.1016/jbbamem.2015.02.010 (2015). PMC4539303 25701232

[b18] Malmsten M. Antimicrobial peptides. Upsala J Med Sci 119, 199–204 (2014). 24758244 10.3109/03009734.2014.899278 PMC4034559

[b19] Maria-Neto S., de Almeida K. C., Macedo M. L. & Franco O. L. Understanding bacterial resistance to antimicrobial peptides: From the surface to deep inside. Biochim Biophys Acta 10.1016/jbbamem.2015.02.017 (2015). 25724815

[b20] Matamouros S. & Miller S. I. S. Typhimurium strategies to resist killing by cationic antimicrobial peptides. Biochim Biophys Acta 10.1016/jbbamem.2015.02.013 (2015). PMC4520786 25644871

[b21] Strange R. N. & Scott P. R. Plant disease: a threat to global food security. Annu Rev Phytopathol. 43, 83–116 (2005). 16078878 10.1146/annurev.phyto.43.113004.133839

[b22] Makovitzki A., Viterbo A., Brotman Y., Chet I. & Shai Y. Inhibition of fungal and bacterial plant pathogens *in vitro* and in planta with ultrashort cationic lipopeptides. Appl Environ Microbiol 73, 6629–36 (2007). 17720828 10.1128/AEM.01334-07 PMC2075073

[b23] Melo M. N. *et al.* Interaction of the Dengue virus fusion peptide with membranes assessed by NMR: The essential role of the envelope protein Trp101 for membrane fusion. J Mol Biol 392, 736–46 (2009). 19619560 10.1016/j.jmb.2009.07.035 PMC7094664

[b24] Bhattacharjya S. De novo designed lipopolysaccharide binding peptides: structure based development of antiendotoxic and antimicrobial drugs. Curr Med Chem 17, 3080–93 (2010). 20629624 10.2174/092986710791959756

[b25] Bhattacharjya S., Domadia P. N., Bhunia A., Malladi S. & David S. A. High-resolution solution structure of a designed peptide bound to lipopolysaccharide: transferred nuclear Overhauser effects, micelle selectivity, and anti-endotoxic activity. Biochemistry 46, 5864–74 (2007). 17469802 10.1021/bi6025159

[b26] Ferguson A. D. *et al.* A conserved structural motif for lipopolysaccharide recognition by procaryotic and eucaryotic proteins. Structure 8, 585–92 (2000). 10873859 10.1016/s0969-2126(00)00143-x

[b27] Deretic V., Dikshit R., Konyecsni W. M., Chakrabarty A. M. & Misra T. K. The algR gene, which regulates mucoidy in Pseudomonas aeruginosa, belongs to a class of environmentally responsive genes. J Bacteriol 171, 1278–83 (1989). 2493441 10.1128/jb.171.3.1278-1283.1989 PMC209741

[b28] Meluleni G. J., Grout M., Evans D. J. & Pier G. B. Mucoid Pseudomonas aeruginosa growing in a biofilm *in vitro* are killed by opsonic antibodies to the mucoid exopolysaccharide capsule but not by antibodies produced during chronic lung infection in cystic fibrosis patients. J Immunol 155, 2029–38 (1995). 7636254

[b29] Rosenfeld Y., Lev N. & Shai Y. Effect of the hydrophobicity to net positive charge ratio on antibacterial and anti-endotoxin activities of structurally similar antimicrobial peptides. Biochemistry 49, 853–61 (2010). 20058937 10.1021/bi900724x

[b30] Jiang Z. *et al.* Effects of net charge and the number of positively charged residues on the biological activity of amphipathic alpha-helical cationic antimicrobial peptides. Biopolymers 90, 369–83 (2008). 18098173 10.1002/bip.20911 PMC2761230

[b31] Bai Y. *et al.* Progressive structuring of a branched antimicrobial peptide on the path to the inner membrane target. J Biol Chem 287, 26606–17 (2012). 22700968 10.1074/jbc.M112.363259 PMC3411001

[b32] Kaconis Y. *et al.* Biophysical mechanisms of endotoxin neutralization by cationic amphiphilic peptides. Biophys J 100, 2652–61 (2011). 21641310 10.1016/j.bpj.2011.04.041 PMC3117184

[b33] Schmidtchen A. & Malmsten M. (Lipo)polysaccharide interactions of antimicrobial peptides. J Colloid Interface Sci (2014) 10.1016/j.jcis.2014.11.024 (2014). 25490856

[b34] Mangoni M. L. *et al.* Lipopolysaccharide, a key molecule involved in the synergism between temporins in inhibiting bacterial growth and in endotoxin neutralization. J Biol Chem 283, 22907–17 (2008). 18550541 10.1074/jbc.M800495200

[b35] Lee D. K. *et al.* Lipid composition-dependent membrane fragmentation and pore-forming mechanisms of membrane disruption by pexiganan (MSI-78). Biochemistry 52, 3254–63 (2013). 23590672 10.1021/bi400087n PMC3795814

[b36] Sciacca M. F. *et al.* Two-step mechanism of membrane disruption by Aβ through membrane fragmentation and pore formation. Biophys J 103, 702–10 (2012). 22947931 10.1016/j.bpj.2012.06.045 PMC3443794

[b37] Ghosh A. *et al.* Sequence context induced antimicrobial activity: insight into lipopolysaccharide permeabilization. Mol Biosyst 10, 1596–612 (2014). 24714742 10.1039/c4mb00111g

[b38] Clore G. M. & Gronenborn A. M. Theory and applications of the transferred nuclear overhauser effect to the study of the conformations of small ligands bound to proteins. J Magn Reson 48, 402–417 (1982).

[b39] Yu L., Tan M., Ho B., Ding J. L. & Wohland T. Determination of critical micelle concentrations and aggregation numbers by fluorescence correlation spectroscopy: aggregation of a lipopolysaccharide. Anal Chim Acta 556, 216–25 (2006). 17723352 10.1016/j.aca.2005.09.008

[b40] Laskowski R. A., Rullmannn J. A., MacArthur M. W., Kaptein R. & Thornton J. M. AQUA and PROCHECK-NMR: programs for checking the quality of protein structures solved by NMR. J Biomol NMR 8, 477–86 (1996). 9008363 10.1007/BF00228148

[b41] Bhunia A. *et al.* NMR structure of pardaxin, a pore-forming antimicrobial peptide, in lipopolysaccharide micelles: mechanism of outer membrane permeabilization. J Biol Chem 285, 3883–95 (2010). 19959835 10.1074/jbc.M109.065672 PMC2823531

[b42] Han X., Bushweller J. H., Cafiso D. S. & Tamm L. K. Membrane structure and fusion-triggering conformational change of the fusion domain from influenza hemagglutinin. Nat Struct Biol 8, 715–20 (2001). 11473264 10.1038/90434

[b43] Zeitler B. *et al.* De-novo design of antimicrobial peptides for plant protection. PLoS One 8, e71687 (2013). 23951222 10.1371/journal.pone.0071687 PMC3741113

[b44] Babalola O. O. Beneficial bacteria of agricultural importance. Biotechnol Lett 32, 1559–70 (2010). 20635120 10.1007/s10529-010-0347-0

[b45] Lugtenberg B. & Kamilova F. Plant-growth-promoting rhizobacteria. Annu Rev Microbiol 63, 541–56 (2009). 19575558 10.1146/annurev.micro.62.081307.162918

[b46] Meindre F. *et al.* The Nuclear Magnetic Resonance Solution Structure of the Synthetic AhPDF1.1b Plant Defensin Evidences the Structural Feature within the γ-Motif. Biochemistry 53, 7745–54 (2014). 25419866 10.1021/bi501285k

[b47] Mirouze M. *et al.* A putative novel role for plant defensins: a defensin from the zinc hyper-accumulating plant, Arabidopsis halleri, confers zinc tolerance. Plant J 47, 329–42 (2006). 16792695 10.1111/j.1365-313X.2006.02788.x

[b48] Lay F. T. & Anderson M. A. Defensins–components of the innate immune system in plants. Curr Protein Pept Sci 6, 85–101 (2005). 15638771 10.2174/1389203053027575

[b49] Dhople V., Krukemeyer A. & Ramamoorthy A. The human beta-defensin-3, an antibacterial peptide with multiple biological functions. Biochim Biophys Acta 1758, 1499–1512 (2006). 16978580 10.1016/j.bbamem.2006.07.007

[b50] Li W. *et al.* Proline-rich antimicrobial peptides: potential therapeutics against antibiotic-resistant bacteria. Amino Acids 46, 2287–94 (2014). 25141976 10.1007/s00726-014-1820-1

[b51] Fernandez D. I., Lee T. H., Sani M. A., Aguilar M. I. & Separovic F. Proline facilitates membrane insertion of the antimicrobial peptide maculatin 1.1 via surface indentation and subsequent lipid disordering. Biophys J 104, 1495–507 (2013). 23561526 10.1016/j.bpj.2013.01.059 PMC3617439

[b52] Castro M. S. & Fontes W. Plant defense and antimicrobial peptides. Protein Pept Lett 12, 13–8 (2005). 15638798

[b53] Montesinos E. & Bardají E. Synthetic antimicrobial peptides as agricultural pesticides for plant-disease control. Chem Biodivers 5, 1225–37 (2008). 18649311 10.1002/cbdv.200890111

[b54] Jan P. S., Huang H. Y. & Chen H. M. Expression of a synthesized gene encoding cationic peptide cecropin B in transgenic tomato plants protects against bacterial diseases. Appl Environ Microbiol 76, 769–75 (2010). 19966019 10.1128/AEM.00698-09 PMC2813020

[b55] Malnoy M., Venisse J. S., Reynoird J. P. & Chevreau E. Activation of three pathogen-inducible promoters of tobacco in transgenic pear (Pyrus communis L.) after abiotic and biotic elicitation. Planta 216, 802–14 (2003). 12624768 10.1007/s00425-002-0932-0

[b56] D M., M I., R T. & A B. Rapid recording of 2D NMR spectra without phase cycling. Application to the study of hydrogen exchange in proteins. J Magn Reson 85, 393–399 (1989).

[b57] V S., M P., R L. & V S. Gradient-tailored water suppression for 1H-15N HSQC experiments optimized to retain full sensitivity. J Magn Reson 102, 241–245 (1993).

[b58] Güntert P., Mumenthaler C. & Wüthrich K. Torsion angle dynamics for NMR structure calculation with the new program DYANA. J Mol Biol 273, 283–98 (1997). 9367762 10.1006/jmbi.1997.1284

[b59] Grewal R. K., Gupta S. & Das S. Xanthomonas oryzae pv oryzae triggers immediate transcriptomic modulations in rice. BMC Genomics 13, 49 (2012). 22289642 10.1186/1471-2164-13-49 PMC3298507

