# Supplementary material for: Antimicrobial Peptides: Insights into Membrane Permeabilization, Lipopolysaccharide Fragmentation and Application in Plant Disease Control
Source: Sci Rep. 2015 Jul 6;5:11951. doi: 10.1038/srep11951 (PMC4491704; doi:10.1038/srep11951)
Supplement: Supplementary Information [file srep11951-s1.doc]

**Supplementary Information**

**Antimicrobial Peptides: Insights into Membrane Permeabilization, Lipopolysaccharide Fragmentation and Application in Plant Disease Control**

Aritreyee Datta^1^, Anirban Ghosh^1^, Cristina Airoldi^2^, Paola Sperandeo^2^, Kamal H. Mroue^3^, Jesus Jimenez-Barbero^4,5^, Pallob Kundu^6^, Ayyalusamy Ramamoorthy,^3^ Anirban Bhunia^1,3^,*

^1^Department of Biophysics, Bose Institute, P-1/12 CIT Scheme, Kolkata 700 054, India

^2^Department of Biotechnology and Biosciences, University of Milano-Bicocca, P.zza della Scienza 2, 2016 Milano, Italy

^3^Biophysics and Department of Chemistry, University of Michigan, 930 N. University Avenue, Ann Arbor, Michigan 48109-1055, USA

^4^Infectious Diseases Program, CIC bioGUNE, Parque Tecnologico de Bizkaia, Building 801A, 48160 Derio, Spain

^5^IKERBASQUE, Basque Foundation for Science, 48011 Bilbao, Spain

^6^Division of Plant Biology, Bose Institute, P-1/12 CIT Scheme, Kolkata 700 054, India

*Corresponding Author:

Anirban Bhunia

Tel: (91) 33 2569 3336

E-mail address: abhunia@umich.edu or bhunia@jcbose.ac.in

**Experimental Procedures**

**Reagents**

*E. coli* O111:B4 LPS, spin labelled lipids 5-doxyl stearic acid, 16-doxyl stearic acid, Polymyxin B and calcein were purchased from Sigma Aldrich Co. (St. Louis, USA). 4, 4-dimethyl-4-silapentane-1-sulfonic acid (DSS), and deuterium oxide were purchased from Cambridge Isotope Laboratories, Inc. (Tewksbury, USA). All peptides were purchased from GL Biochem (Shanghai, China) with 95% purity.

**Bacterial and Fungal Strains**

Common laboratory strain *E.coli* DH5α, *Pseudomonas aeruginosa* (ATCC 27853) and *Bacillus subtilis* (MTCC) were obtained either from ATCC (USA) or MTCC (India). Plant pathogenic strains *Xanthomonas campestris* pv. *Campestris*, (isolated locally from fields of Kalyani, West Bengal, India), *Xanthomonas oryzae pv. oryzae* (kindly provided by Professor Sampa Das, Bose Institute, India) and fungal strains *Candida albicans* SC5314, *Cryptococcus neoformans var. grubii* H99 were used in this study.

**Plant Materials and Culture**

Grains of rice (*Oryza sativa*) variety IR64 were surface sterilized using 5% HgCl_2_, washed thoroughly with distilled water to remove all traces of HgCl_2_, and planted in soil rite. The plants were grown in normal light at 30 ⁰C. Cabbage (*Brassica oleracea*) seeds variety VC612 and Golden Acre obtained from Sutton Seeds, India, were soaked overnight in water, planted in Soil rite (Keltech, India) and grown in a growth chamber at 10000 lux, 25 ⁰C, 85% relative humidity and a photoperiod of 16 hours.

**Media composition:**

For antimicrobial activity assay, *E.coli, Bacillus* *subtilis* and *Pseudomonas aeruginosa* were grown in Mueller Hinton broth, *X. campestris pv. campestris* in PSP broth (0.5% peptone, 2% sucrose dissolved in starch obtained from boiling 200 g of potato/litre of media), *X. oryzae* In PS broth (1% peptone, 1% sucrose), and *C. albicans* and *C. grubii* in YPDU broth (1% yeast extract, 1% peptone, 2% dextrose and 20µg/mL uridine)

**Antimicrobial activity**

Antimicrobial activity was tested using a modified version of a standard microdilution broth assay as described previously[^1^](#_ENREF_1). Mid log phase cultures of *E. coli* DH5α, *X. campestris* pv. *Campestris*, *X. oryzae*, *B. subtilis*, *P. aeruginosa*, *C. albicans* and *C. grubii* were obtained by inoculating with overnight grown cultures of respective pathogens in their respective growth medium. The cell suspensions were centrifuged at 6000 rpm for 10 minutes. Cell pellets were washed thrice with 10 mM phosphate buffer of pH 7.4 and re-suspended in the same buffer to obtain a bacterial cell suspension containing 10^5^ CFU/mL and a fungal suspension of 10^4^ CFU/mL. In a 96 well plate format, 50 µL of this suspension was incubated with different concentrations of peptide (ranging from 1 µM to 100 µM) prepared from 1 mM peptide stock in phosphate buffer (pH 7.4) and incubated at 37 ᵒC for 3 to 6 hours. A positive control containing only cell suspension and a negative control containing 10 µM Polymyxin B with cell suspension was maintained. 150 µL of suitable media was added to each well and incubated overnight without shaking at 37 °C. Absorbance of the culture was read at 600 nm to monitor bacterial growth. The peptide concentration at which no growth was observed served as its MIC (minimum inhibitory concentration). All experiments were performed in triplicates.

**Neutralisation of Endotoxin**

The ability of the designed peptides to neutralize was estimated using a Novagen Chromogenic Limulus amoebocyte lysate (LAL) assay kit using a modified version of a previously published protocol[^2^](#_ENREF_2). The kit instructions were followed to perform the test. Briefly, 1, 5, 10, 15, and 25 µM of the peptides were prepared from peptide stocks in pyrogen-free water, incubated with 0.5, and 1 EU (Endotoxin unit)/mL of *E. coli* O55:B5 LPS (1 EU =0.13 ng of LPS), in a final volume of 100 µL, at 37 °C for 30 min. The mixture was further incubated for another two hours after addition of an equal volume of LAL followed by addition of chromogenic substrate. Color development is a characteristic of a positive reaction that indicates the presence of endotoxin. The absorbance was measured at 405 nm to detect the neutralization of endotoxin by AMP. Average values obtained from triplicates have been reported.

**Live-cell NMR Experiments**

*E. coli* DH5α cultures were grown at 37 °C in LD medium to mid-logarithmic phase (OD_600_=0.4 – 0.6) . Bacterial cells were washed four times in 10 mM sodium phosphate buffer (pH 7.2) by centrifuging 10 min at 7000 rpm and finally resuspended in the same buffer to a final concentration of approximately 5 × 10^9^ cells/ml. The number of living cells before and after peptide addition was evaluated by plating serial dilutions of the sample onto solid medium and incubating at 37 °C for 16-18 hours. Depending on the desired final number of cells, the corresponding amount of cell suspension was taken. The suspension was then split into two aliquots of the same volume and centrifuged for 5 min at 7000 rpm to remove the supernatant and obtain two pellets containing approximately the same number of living cells. The first pellet was suspended in 10 mM sodium phosphate buffer (pH 7.2), while the second was suspended in the same buffer but containing the peptide at the final desired concentration. All NMR experiments were performed on a Bruker Avance III 600 MHz NMR spectrometer, equipped with a 5 mm QCI cryoprobe. Each sample (total volume 550 μL) was transferred into a 5 mm NMR tube and maintained at a temperature of 310 K during experiments. Final peptide concentration was in the range of 0.5-1.5 mM, with a total cell number varying from 3×10^8^ to 2×10^9^. For all live-cell NMR experiments, a recycle delay of 2s and an acquisition time of 2s were used. The total duration of each experiment was ~ 5 min.

**Fluorescence spectroscopy**

Binding studies of peptide with LPS were performed using various fluorescence spectroscopy experiments. The intrinsic Trp fluorescence emission spectra of 5 µM peptide upon titration with increasing concentrations of LPS (ranging from 0 to 10 µM) was measured at an emission range of 300-400 nm using an excitation wave length of 280 nm, and excitation and emission slit of 5 nm. All the fluorescence experiments were performed at 25 ᵒC in a quartz cuvette of path length 0.1 cm using a Hitachi F-7000 FL spectrometer. Peptide and LPS stocks were prepared in 10 mM Phosphate buffer of pH 6.0. Further, addition of a quencher acrylamide, in increasing concentrations (0-0.5 M) to both free and bound peptide yielded Stern-Volmer’s quenching constant (Ksv) for both states, calculated using the following equation[^5^](#_ENREF_5):

**F_0_/F = 1+Ksv [Q]**  **Equation (1)**

where F_0_= fluorescence intensity in absence of quencher, F= fluorescence intensity in presence of quencher at each titration, [Q] is the molar concentration of the quencher[^5^](#_ENREF_5). Steady-state anisotropy experiments were also recorded for 5 µM peptide upon titration with increasing concentrations of LPS up to 10 µM using a polarization accessory. Vertically polarized light at 280 nm was used for excitation with a slit of 5 nm and Anisotropy (r) values of intrinsic Trp fluorescence were obtained using the equation[^5^](#_ENREF_5):

**r = (I_VV_ – G*I_VH_) / (I_VV_ + 2*G*I_VH_)** **Equation (2)**

where G is the sensitivity factor of the instrument, I_VV_ and I_VH_ are the vertically and horizontally polarized components of probe, respectively[^5^](#_ENREF_5).

**Depth of insertion assay**

Fluorescence-quenching studies were carried out using two spin-labelled lipids, 5-DSA (shallow quencher) and 16-DSA (deep quencher) to ascertain the depth of insertion of Trp residue into the hydrophobic core of LPS. Stock solutions of the lipids in methanol were added to 5 µM peptide and 40 µM LPS vesicles. The position of Trp in the LPS bilayer (Z_cf_) was calculated using Parallax analysis method from the equation[^6^](#_ENREF_6):

**Z_cf_ = L_c1_+[(-1/π C ln(F_1_/F_2_)-L_21_^2^]/2L_21_** **Equation (3)**

where L_c1_ is the difference in depth between the shallow quencher and the bilayer center, F_1_ and F_2_ are the relative Trp fluorescence intensities in the presence of the shallow and deeper quencher, respectively. L_21_ is the difference in depth between the shallow and deeper quencher, C is the mole fraction of quencher per unit area assuming the surface area of the LPS bilayer to be 70 Å[^6^](#_ENREF_6).

**Dynamic Light Scattering (DLS)**

Malvern ZetasizerNano S (Malvern Instruments, UK) provided with a 4-mW He-Ne laser (λ= 633 nm) and a back scattering angle of 173° was used for all experiments. Peptide and LPS at a molar ratio of 20:1 were dissolved in 10 mM phosphate buffer; pH 7.4, filtered using Millipore 0.45-μm polycarbonate membrane filters, degassed before use and measured at 298 K in low volume disposable sizing cuvettes. For data analysis, the viscosity and refractive index of 10 mM phosphate buffer were taken to be 0.89 and 1.33, respectively.

**Hemolytic assay**

Blood cell pellet, obtained from fresh human blood by centrifugation at 4000 × g for 10 min at 4 ⁰C was washed thrice with PBS (pH 7.4) and resuspended to obtain a 1×10^9^ erythrocytes/mL suspension. The above suspension was incubated with equal volumes of increasing concentrations of VG16KRKP up to 250 µM at 37 ⁰C in shaking condition for 1 hour. Samples were again centrifuged at 900 × g for 10 min at 4 ⁰C and the absorbance of supernatant was measured at 540 nm to quantify RBC lysis. 2% Triton X 100 was used as a control which caused complete (100%) lysis. Percentage hemolysis of VG16KRKP was calculated using the equation:

**Percentage of hemolysis = (O_p_-O_b_)/(O_m_-O_b_) × 100 Equation (4)**

where O_p_ is the optical density of the sample upon treatment with a given peptide concentration, O_b_ is the optical density of buffer and O_m_ is the optical density of Triton X 100.

***In vitro* cytotoxicity assay**

Cytotoxicity assay was conducted on HT1080, a human fibrosarcoma cell line obtained from ATCC, maintained in Dulbecco's modified Eagle's medium (DMEM) containing Penicillin, Streptomycin, Gentamycin and Amphotericin B. Cells from a frozen stock were spread on a 100-mm dish in DMEM and incubated for 24 hr at 37 °C, 5% CO_2_, 100 % relative humidity. The 0.02 % trypsin was used to bring the cells into suspension followed by PBS wash and re-suspension in DMEM. A 24-well plate culture was set up with 500 µL of cell suspension at a starting confluence of 30 % and allowed to grow till 50 %. Increasing concentrations of VG16KRKP was added to each of the wells and incubated at 37 °C, 5% CO_2_ for 44 hours. A control sample containing only cells was maintained. The maximum concentration of peptide used was 50 µM. The 0.5 % Triton X 100 was used as a positive control that caused 80 % cell death. Cells were stained with 100 µL of methylene blue solution (1 % in 50 % methanol) by incubation at room temperature for 30 min. The stain was aspirated, plates rinsed with Millipore water twice and air-dried. Cell numbers were determined by scanning the plate at 620 nm on a Thermo Scientific Varioskan Flash multimode microplate reader. An average of 50 absorbance readings taken at different points on each well was measured. Percentage viability of cells was calculated using the equation:

**Percentage viability = (O.D of treated cells/O.D of untreated control cells) × 100 Equation (5)**

**Table S1**. Long range NOEs (i, ≥ i+5) used to determine the three-dimensional structure of VG16KRKP in LPS.

| **VG16KRKP in LPS** |
| --- |
| Trp5C^α^H-Leu11HN  Trp5C^α^H-Phe12H3  Trp5C^β^Hs-Phe12HN  Trp5C^β^Hs-Phe12H2  Pro10C^γ^Hs-Trp5H6  Leu11C^β^Hs-Trp5H6  Leu11C^δ^Hs-Trp5H6  Leu11C^δ^Hs-Trp5H2  Phe12C^β^Hs-Trp5HN  Phe12C^β^Hs-Trp5H2  Lys14 C^α^H-Leu11HN |


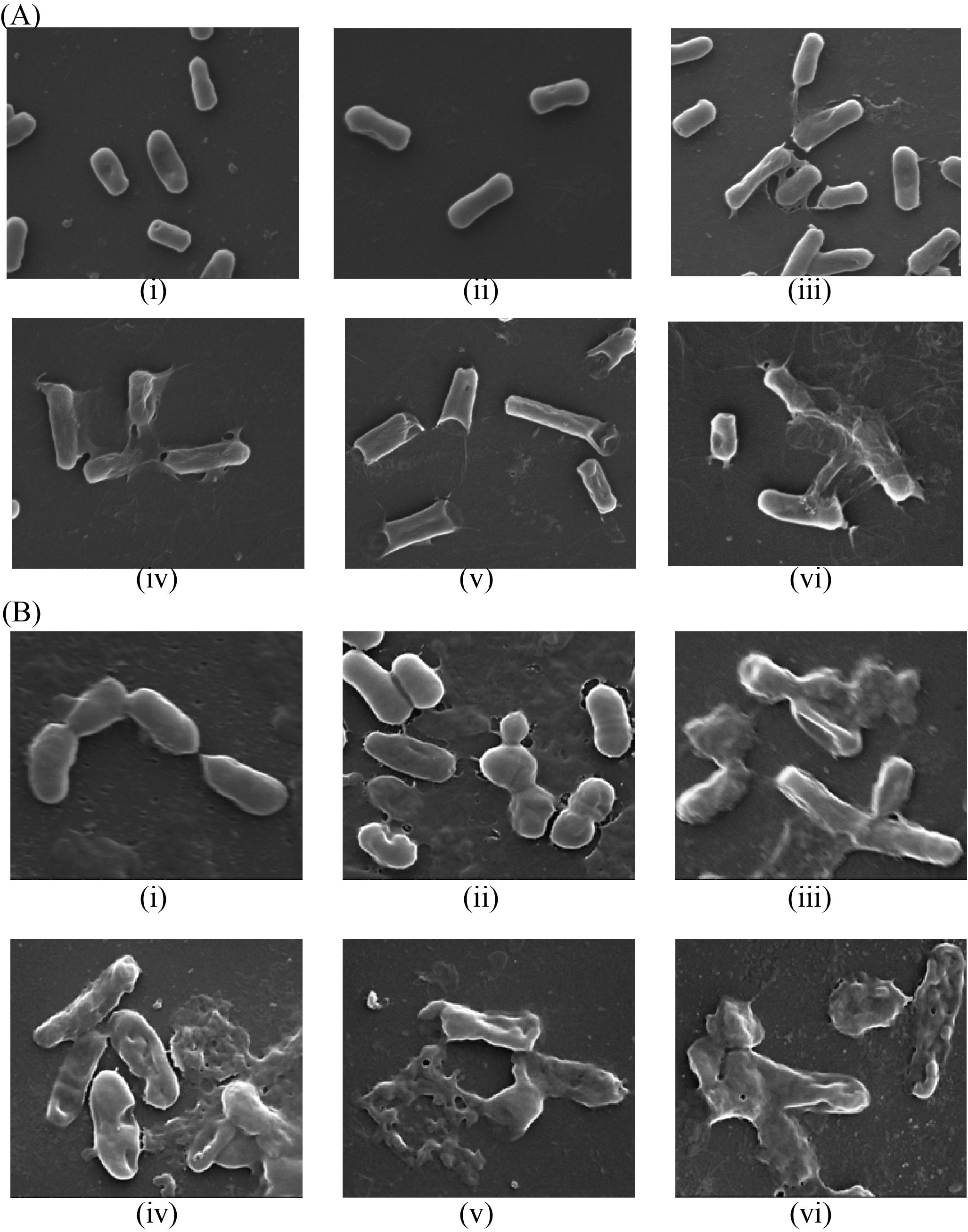


**Figure S1. Scanning electron microscopy (SEM) images of *E. coli* and *X. oryzae* pre- and post-treatment with VG16KRKP at different time points.** (A) SEM images of untreated *E. coli* control sample at (i) 0 min, and (ii) 45 min; *E. coli* treated with 10 µM VG16KRKP after (iii) 5 min, (iv) 15 min, (v) 30 min and (vi) 45 min. (B) SEM images of untreated *X. oryzae* control sample at (i) 0 min, and (ii), 45min; *X. oryzae* treated with 15 µM VG16KRKP after (iii) 5 min, (iv) 15 min, (v) 30 min and (vi) 45 min. Both cell types show initiation of membrane lysis from as early as 5 min post treatment with VG16KRKP at their respective MICs. All images were taken at 25× magnifications.


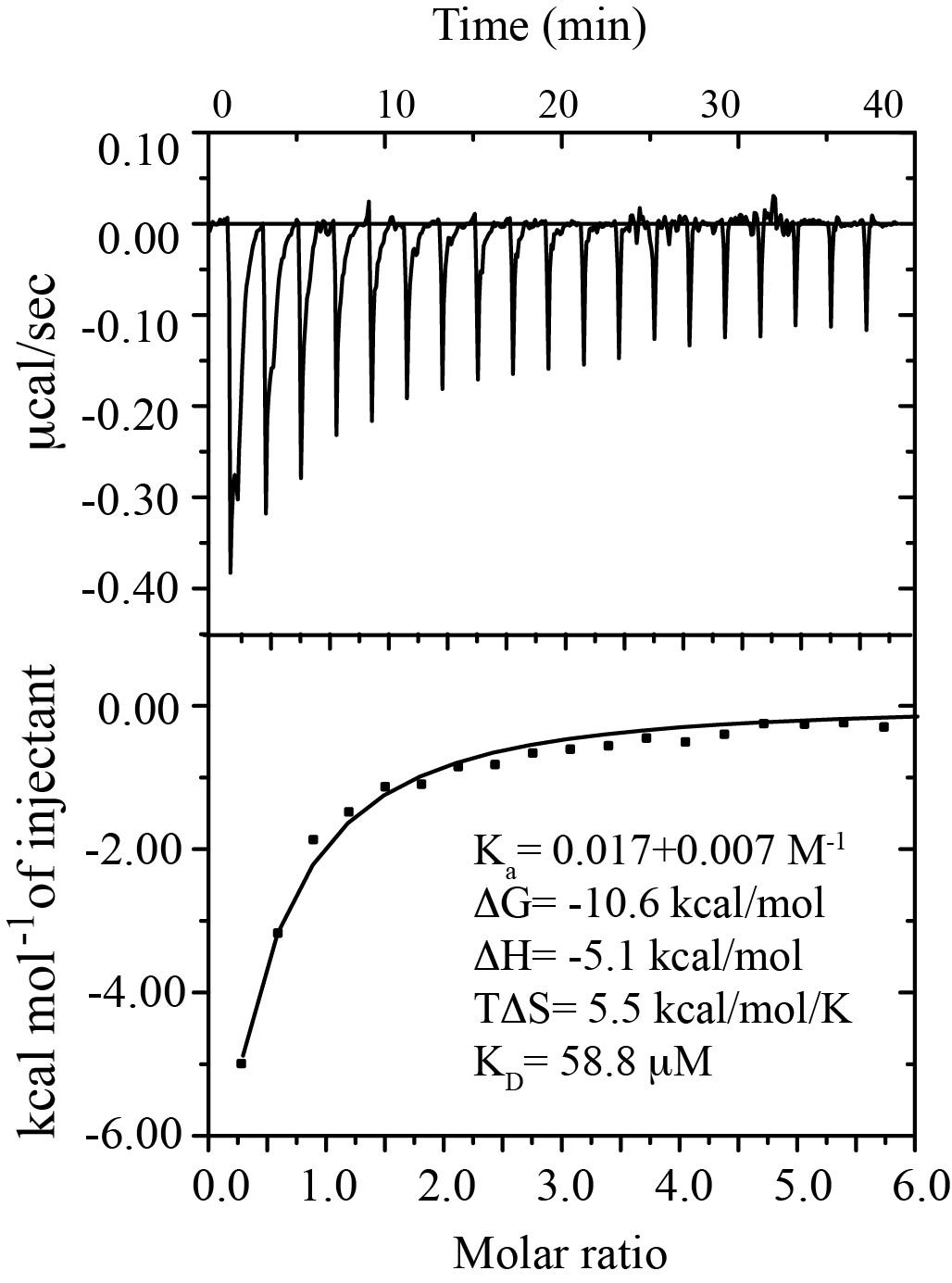


**Figure S2. Binding studies of VG16A**. Upper panel showing the endothermic heat of reaction vs. time (in minutes) upon interaction of VG16A with LPS. The lower panel of the figure shows enthalpy change per mole of peptide injection vs. molar ratio (peptide:LPS) for VG16A. 50 µM of LPS was titrated against 200 µM of peptide for this experiment.

**
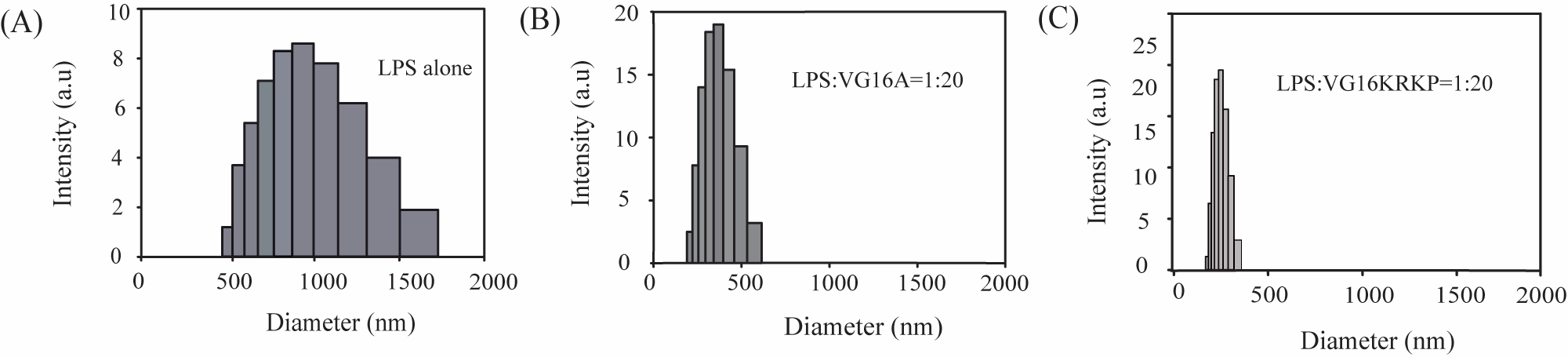
**

**Figure S3. Disaggregation studies of LPS by VG16A and VG16KRKP.** Bar diagrams showing hydrodynamic diameter (nm) versus intensity (arbitrary unit, AU) of scattered light for (A) LPS and in the presence of (B) VG16A and (C) VG16KRKP. All the experiments were performed using 10 mM sodium phosphate buffer (pH 7.4) at 298 K. DLS measurements show the disaggregation of LPS micelle in the presence of VG16KRKP to a much greater extent than VG16A.

**
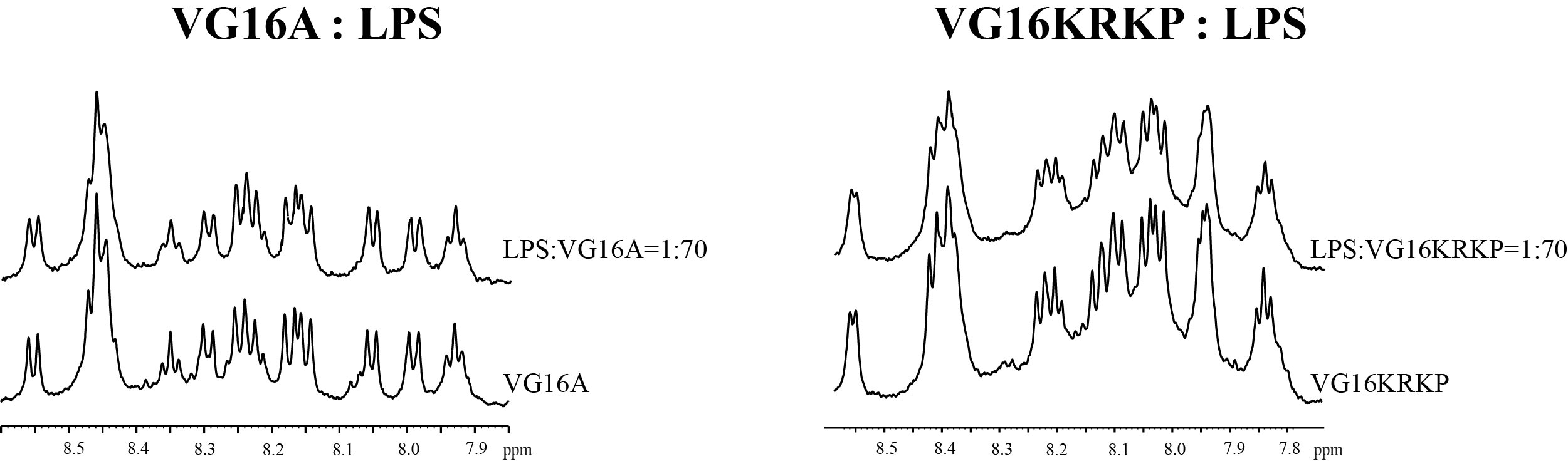
**

**Figure S4. Titration.** One-dimensional ^1^H NMR spectra of VG16A and VG16KRKP in the absence and presence of LPS, respectively. The spectra showing the line broadening effect of the peptides in the presence of LPS. Experiments were performed at 298 K on a 500-MHz Bruker Avance III NMR spectrometer.

**
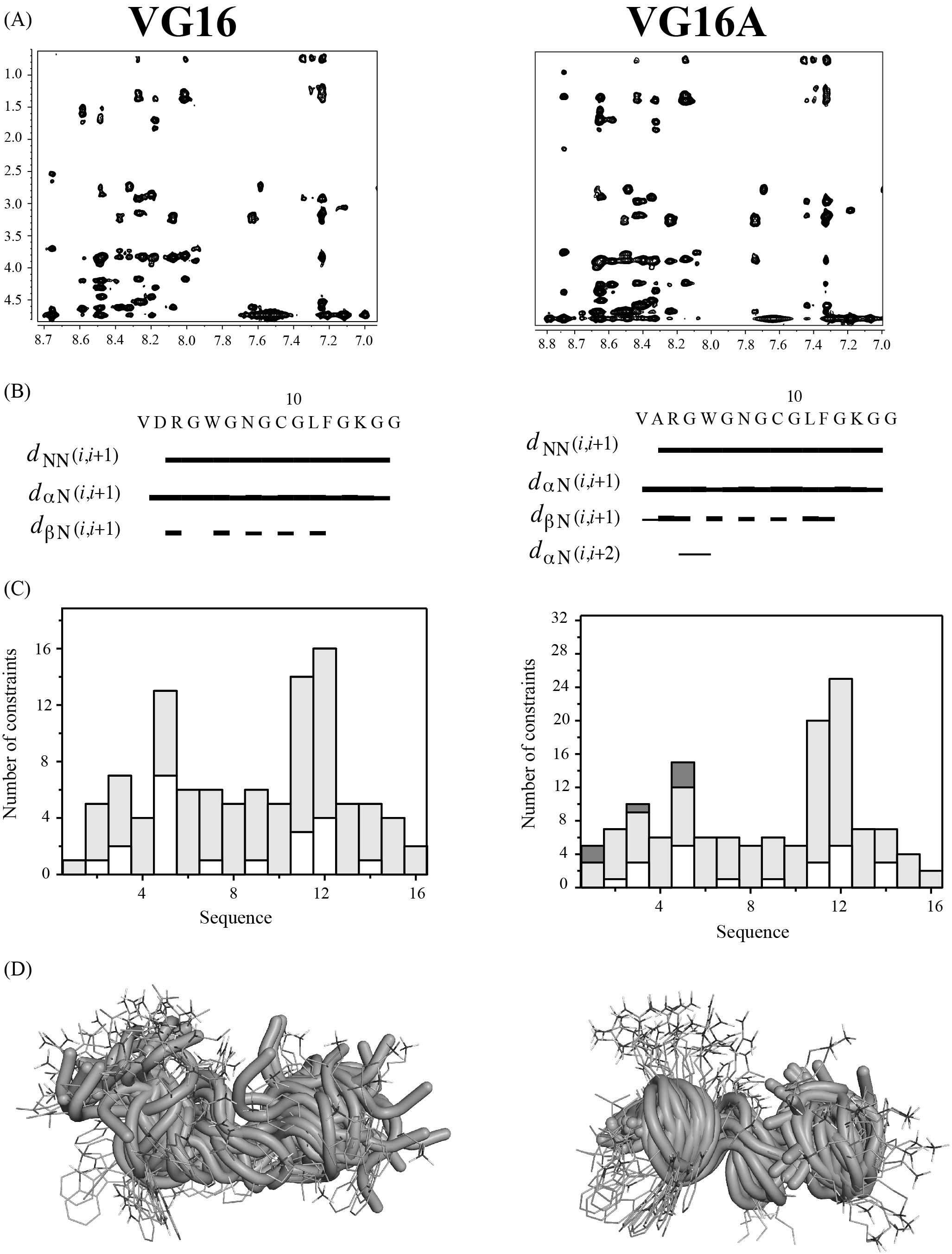
**

**Figure S5. Two-dimensional trNOESY NMR spectra and NMR structural parameters of VG16 and VG16A in LPS**. (A) ^1^H-^1^H two-dimensional trNOESY spectra of VG16 and VG16A showing only intra-residual and sequential NOE contacts. (B) Bar diagram summarizing type (sequential, medium range, and long-range) and number of NOE contacts, in the trNOESY spectra of VG16 and VG16A in LPS. The thickness of the bars indicates the intensity of the NOESY peaks, which are assigned as strong, medium, and weak. The primary amino acid sequences of each peptide are shown at the top. (C) Histogram showing the number and type (intra, sequential, medium) of trNOEs of VG16 and VG16A as a function of residue number in LPS (D) Ensemble of twenty lowest energy structures of VG16 and VG16A showing a random coil structure.


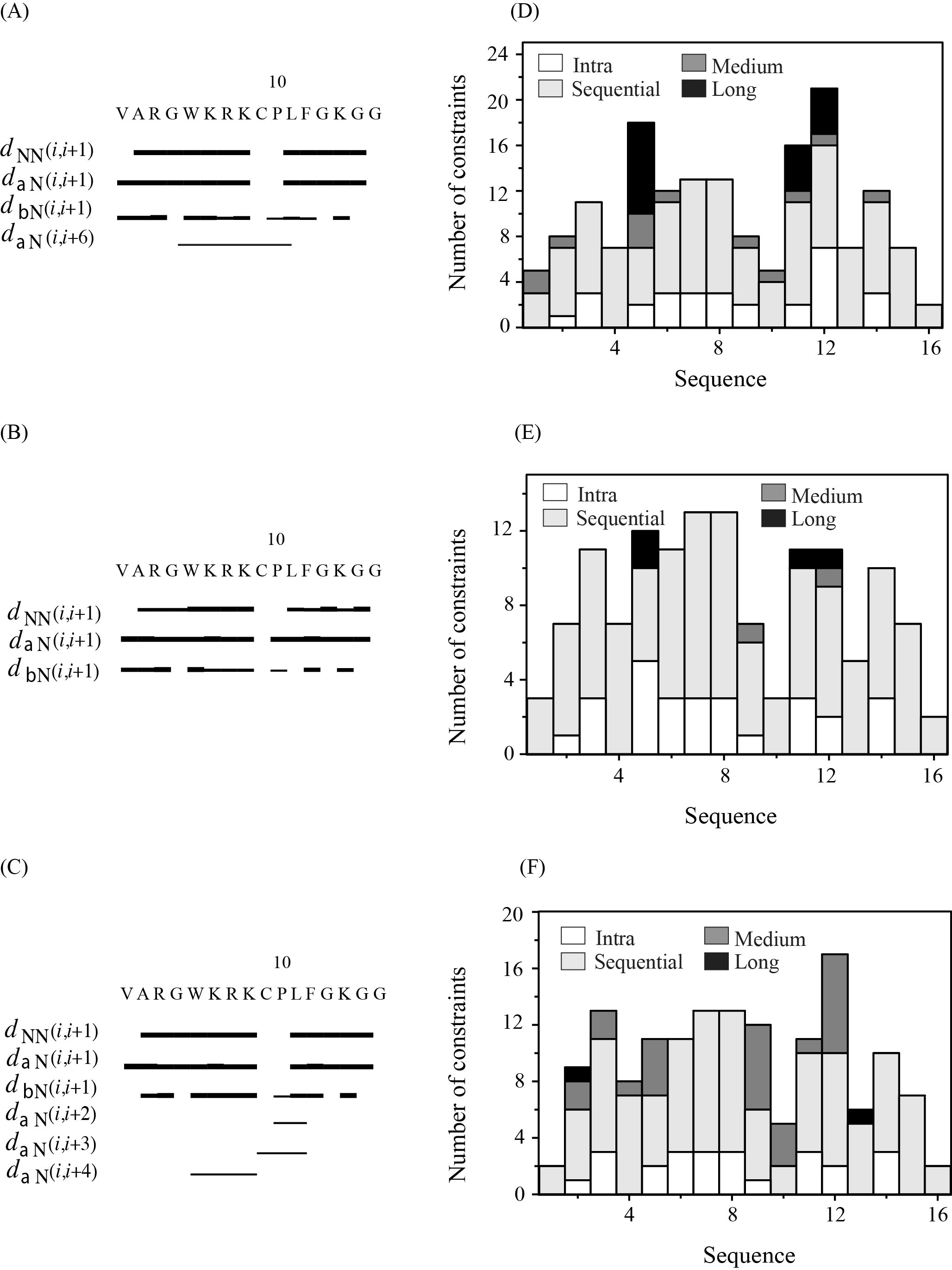


**Figure S6. NMR structural parameters of VG16KRKP in LPS.** (Left panel) Bar diagram summarizing the sequential (i, i), medium range (i, i+2/i+3/i+4), long-range (i, >i+5) and (right panel) number of NOE contacts in the trNOESY spectra of VG16KRKP in LPS. The thickness of the bars indicates the intensity of the NOESY peaks, which are assigned as strong, medium, and weak. The primary amino acid sequences of each peptide are shown at the top. (Right panel) Histogram showing the number and type (intra, sequential, medium) of trNOEs of VG16KRKP as a function of residue number in LPS.

**
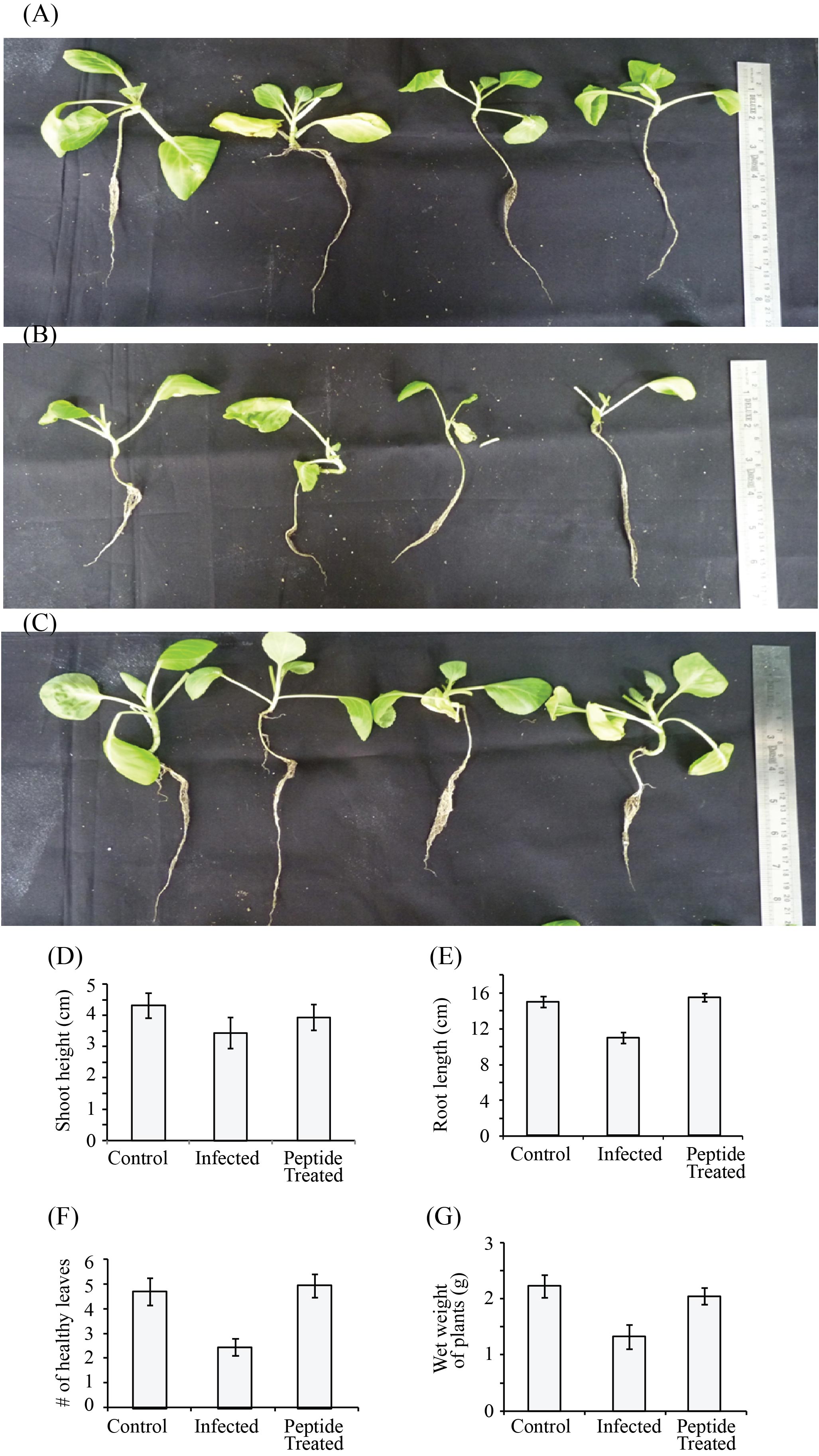
**

**Figure S7. Application of VG16KRKP for treating *X. campestris* infection in cabbage plants.** (A) Images of 50-days old uprooted control, (B) infected, and (C) peptide-treated cabbage plants showing inhibition of disease upon treatment. (D) Bar plots showing variation in shoot height, (E) root length, (F) Number of healthy leaves, and (G) wet weight of plants, between control, infected and peptide treated cabbage plants which signify that VG16KRKP is capable of inhibiting disease to a significant extent. A students “t”-test to determine the statistical significance of the difference between the infected and the treated plants for all the tested parameters yielded a p value <0.05 in each case.

**References**

1. Blazyk, J. et al. A novel linear amphipathic beta-sheet cationic antimicrobial peptide with enhanced selectivity for bacterial lipids. *J Biol Chem* **276**, 27899-906 (2001).

2. Bhunia, A., Mohanram, H., Domadia, P.N., Torres, J. & Bhattacharjya, S. Designed beta-boomerang antiendotoxic and antimicrobial peptides: structures and activities in lipopolysaccharide. *J Biol Chem* **284**, 21991-2004 (2009).

3. Hanahan, D. Studies on transformation of Escherichia coli with plasmids. *J Mol Biol* **166**, 557-80 (1983).

4. Sabbattini, P., Forti, F., Ghisotti, D. & Dehò, G. Control of transcription termination by an RNA factor in bacteriophage P4 immunity: identification of the target sites. *J Bacteriol* **177**, 1425-34 (1995).

5. Lakowicz, R., J., Masters & R., B. *Principles of fluorescence spectroscopy*, (Plenum press, New York, 1983).

6. Chattopadhyay, A. & London, E. Parallax method for direct measurement of membrane penetration depth utilizing fluorescence quenching by spin-labeled phospholipids. *Biochemistry* **26**, 39-45 (1987).

7. Saravanan, R., Bhunia, A. & Bhattacharjya, S. Micelle-bound structures and dynamics of the hinge deleted analog of melittin and its diastereomer: implications in cell selective lysis by D-amino acid containing antimicrobial peptides. *Biochim Biophys Acta* **1798**, 128-39 (2010).

8. Ferre, R. et al. Inhibition of plant-pathogenic bacteria by short synthetic cecropin A-melittin hybrid peptides. *Appl Environ Microbiol* **72**, 3302-8 (2006).
